# Genome-wide analysis of genes encoding core components of the ubiquitin system during cerebral cortex development

**DOI:** 10.1186/s13041-022-00958-z

**Published:** 2022-08-16

**Authors:** Alexandre Bouron, Marie-Odile Fauvarque

**Affiliations:** 1 Université Grenoble Alpes, Inserm, CEA, UMR 1292, 38000 Grenoble, France; 2 Genetics and Chemogenomics Lab, Building C3, CEA, 17 rue des Martyrs, 38054 Grenoble Cedex 9, France

**Keywords:** Rodent, Brain, Cerebral cortex, Ubiquitin, Ubiquitination, Deubiquitinating enzymes

## Abstract

Ubiquitination involves three types of enzymes (E1, E2, and E3) that sequentially attach ubiquitin (Ub) to target proteins. This posttranslational modification controls key cellular processes, such as the degradation, endocytosis, subcellular localization and activity of proteins. Ubiquitination, which can be reversed by deubiquitinating enzymes (DUBs), plays important roles during brain development. Furthermore, deregulation of the Ub system is linked to the pathogenesis of various diseases, including neurodegenerative disorders. We used a publicly available RNA-seq database to perform an extensive genome-wide gene expression analysis of the core components of the ubiquitination machinery, covering Ub genes as well as E1, E2, E3 and DUB genes. The ubiquitination network was governed by only *Uba1* and *Ube2m*, the predominant E1 and E2 genes, respectively; their expression was positively regulated during cortical formation. The principal genes encoding HECT (homologous to the E6-AP carboxyl terminus), RBR (RING-in-between-RING), and RING (really interesting new gene) E3 Ub ligases were also highly regulated. *Pja1*, *Dtx3* (RING ligases) and *Stub1* (U-box RING) were the most highly expressed E3 Ub ligase genes and displayed distinct developmental expression patterns. Moreover, more than 80 DUB genes were expressed during corticogenesis, with two prominent genes, *Uch-l1* and *Usp22,* showing highly upregulated expression. Several components of the Ub system overexpressed in cancers were also highly expressed in the cerebral cortex under conditions not related to tumour formation or progression. Altogether, this work provides an in-depth overview of transcriptomic changes during embryonic formation of the cerebral cortex. The data also offer new insight into the characterization of the Ub system and may contribute to a better understanding of its involvement in the pathogenesis of neurodevelopmental disorders.

**Supplementary Information:**

The online version contains supplementary material available at 10.1186/s13041-022-00958-z.

## Introduction

Ubiquitination is a multistep process during which ubiquitin (Ub), a versatile and highly conserved 76 amino-acid polypeptide, is covalently conjugated to target substrates. It is one of the most common posttranslational modifications of proteins [1] and requires the sequential action of three types of enzymes: Ub-activating (E1) enzymes, Ub-conjugating (E2) enzymes and Ub-ligases (E3) [2]. Ubiquitination is counterbalanced by the action of deubiquitinating enzymes (or deubiquitinases, DUBs) that can reverse the conjugation of Ub to substrates. Mammalian DUBs are classified into seven categories: Ub-specific proteases (USPs), Ub carboxyl-terminal hydrolases (UCHs), otubain proteases (OTUs), Machado-Joseph disease protein domain proteases (MJDs or Josephins), JAB1/MPN/Mov34 metallopeptidases (JAMMs), motif-interacting with Ub-containing novel DUB family (MINDY) and ZUP1 [3, 4]. Ubiquitination is described as a quality control system devoted to protein homeostasis since it targets damaged or misfolded proteins for degradation via the Ub–proteasome system. However, ubiquitination shows a wider physiological importance because the conjugation of Ub can modify the activity of their targets, changing their subcellular localization or involvement in the formation of multiple protein complexes. Therefore, ubiquitination is involved in the regulation of various key cellular processes, such as endocytosis, cell signalling, autophagy and DNA repair [5].

A large number of proteins in the brain are ubiquitinated. For instance, an analysis of the ubiquitome (ubiquitinated proteins) revealed 921 targets in the rat brain with numerous pre- and postsynaptic actors [6]. The Ub pathway controls multiple neuronal processes, such as neuron migration, growth and synaptic transmission [7–10]. The Ub system also governs fundamental mechanisms controlling memory reorganization [11]. Moreover, alterations of the Ub pathway are thought to contribute to neurodevelopmental, cognitive and age-related neurodegenerative diseases [8, 12]. It is thus of paramount importance to understand how the Ub system participates in normal brain formation and development. The aim of this study was to provide an extensive and detailed overview of the expression pattern of genes encoding major factors mediating ubiquitination and deubiquitination during the formation of the cerebral cortex in mice. The data presented rely on a published RNA-seq database [13] covering 4 stages of cortical development corresponding to the beginning (embryonic Day 11, E11), the peak (E13), and the end of neurogenesis (E17), followed by the beginning of the maturation process and neuronal circuit assembly (postnatal Day 1, PN1) [14, 15]. These four periods cover stages of profound cell division (E11-E13) followed by stages characterized by the growth and morphological differentiation of the postmitotic neurons and the establishment of neural networks (E17-PN1) [14, 15]. The analysis of gene expression patterns during development will help to understand the extraordinary complexity of the Ub conjugation/deconjugation system. This study not only presents a description of transcription profile changes during embryonic cerebral cortex formation but also provides an in-depth overview of the core components of the Ub system as discerned through published data.

### Materials and methods

The analysis was based on a published RNA-seq gene expression dataset on E11, E13, E17 and PN1 [13], reflecting the period of cerebral cortex formation in the mouse brain. The complete dataset is freely accessible from the GEO repository with the accession number GSE154677. Throughout this study, the results are expressed in transcripts per million (TPM) and the mean ± standard error of mean (SEM).

## Results and discussion

The data obtained from the genome-scale profiling of gene expression are organized into two parts: the first part covers the ubiquitination process, and the second part is devoted to the genes encoding DUBs, a class of enzymes participating in the novo synthesis of Ub and are responsible of the deubiquitination of substrates.

### Ubiquitination

This first section is subdivided into 4 sections covering the following topics: (1) genes involved in the synthesis of Ub and Ub-like proteins, followed by the genes encoding (2) Ub-activating (E1) enzymes; (3) Ub-conjugating (E2) enzymes; and (4) Ub ligases (E3).

#### Ubiquitin genes

**Fig. 1:**
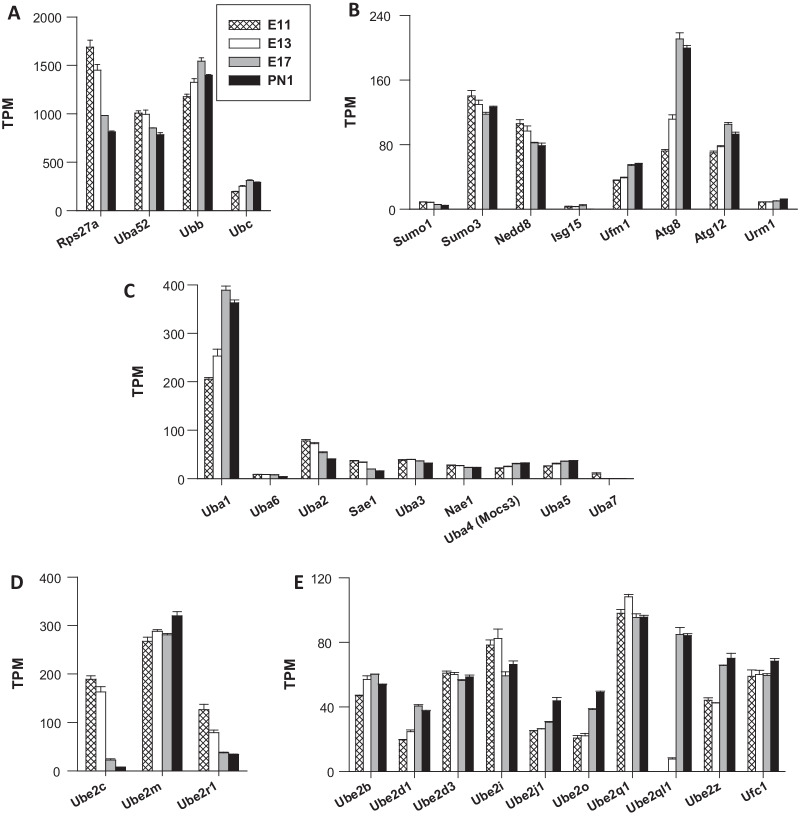
Expression of genes involved in the synthesis of Ub and Ub-like proteins and the genes encoding E1 and E2 enzymes. For this report, the data shown were extracted from datasets derived by genome-wide transcriptome sequencing (RNA-Seq) [13]. The original database is freely available on the GEO repository (www.ncbi.nlm.nih.gov/geo/) with the accession number GSE154677. The analysis covers the temporal pattern of expression of the major genes in the Ub system. RNA was extracted from the cerebral cortex of mice in four stages: embryonic Days 11 (E11), 13 (E13), and 17 (E17) and postnatal Day 1 (PN1). RNA abundance is reported in transcripts per million (TPM), and only genes with TPM values ≥ 2 were considered to be actively transcribed, following the criteria presented in [25]. Figure shows the expression of genes involved in the synthesis of Ub (**A**), Ub-like proteins (**B**), and genes encoding Ub-activating E1 enzymes (**C**) and Ub-conjugating E2 enzymes (**D**,** E**)

In mammals, two classes of genes are involved in de novo synthesis of Ub: the monomeric Ub-ribosomal fusion genes *Uba52* and *Rps27a* (*Uba80*) and the stress-inducible poly-Ub genes *Ubb* and *Ubc* [16]. In the immature cerebral cortex, *Ubb* and *Rps27a* were the most highly expressed Ub genes (Fig. 1A). *Ubb* transcripts accounted for 30–40% of the total Ub transcripts, which is actually in close agreement with previously reported data [17], indicating a high abundance of total *Ubb* transcripts in the brain. *Ubb* and *Rps27a* followed dissimilar expression patterns: transcripts of *Rps27a* predominated at the onset of corticogenesis (E11–13) before their number significantly decreased, whereas the *Ubb* gene was the most highly Ub gene expressed at the end of corticogenesis (E17-PN1). Notably, the number of transcripts (expressed in transcripts per million, TPM) was on the order of 700–1700, reflecting a very high abundance of *Ubb*, *Rps27a* and *Uba52* transcripts at all stages (Fig. 1A). In comparison, the TPM values of *H2afz* (encoding histone 2A, member z), one of the most abundant cellular proteins ubiquitously expressed [18], were 1740 (at E11) and 400 (on PN1). *H2af* was the 66th most highly expressed gene on E11, whereas *Rps27a* was the 68th mostly highly expressed. On PN1, *Ubb*, the most prominent Ub gene (with TPM values of 1400) was the 60th most highly expressed gene in the immature cerebral cortex. These data are in line with the known high abundance of the Ub protein in biological samples, in which it has been shown to comprise up to 5% of total protein [19]. These 4 genes generate single (*Uba52* and *Rps27a*) or multiple copies (9 for *Ubc*, and 3 for *Ubb*) of Ub. Therefore, to better assess the contribution of these genes to the total pool of Ub, we calculated the theoretical production of Ub molecules assuming a similar translation efficiency for the four transcripts [20]. The *Ubc* gene accounted for nearly 65% of the total pool of Ub in the embryonic cortex, followed by *Ubb, Rps27a* and *Uba52*, accounting for 18%, 11% and 6%, respectively. These data are in line with a previous report showing that UBC accounts for 64% of the total Ub pool in HeLa cells [20]. Thus, the poly-ubiquitin gene *Ubc* is the major cellular contributor of Ub molecules.

In the mouse brain, 60% of the Ub pool is in the free form (i.e., not attached to target substrates) [19]. The level of free Ub is important to neuronal functions and survival [16, 19]. The morphology, neurite outgrowth and synaptic development are impaired in cultured neurons isolated from the brains of *Ubb*-knockout mice [21].

A recent study reported 52 Ub pseudogenes in humans [22]. Moreover, some of these genes, such as human *Ubb* pseudogene 4 (*Ubbp4*), *Rps27a* pseudogene 16 (*Rps27ap16*), and *Uba52* pseudogene 8, encode proteins [22]. Here, the expression of the following murine Ub pseudogenes was examined: *Gm1821* (*Ubb-pseudo gene* or *Ubb-ps*), *Rps27a-ps1*, *Rps27a-ps2* and *Gm7866* (*Uba52-ps*). Only transcripts of the pseudogene *Gm1821* were found with TPM values of ⁓ 15, which was nearly ⁓ 100-fold less abundant than *Ubb* transcripts. In conclusion, our data indicated a high expression level of the three Ub-encoding genes *Ubb*, *Rps27a* and *Uba52*. In comparison, the expression of Ub pseudogenes was negligible.

#### Ubiquitin-like genes

Similar to Ub, various Ub-like proteins can be covalently conjugated to target substrates via an enzymatic cascade involving E1, E2, and E3 enzymes [23], [24]. The expression of this set of genes was analysed, and the results are reported in Fig. 1B. The following 10 genes were identified: *Sumo1-3, Nedd8, Isg15, Ubd (Fat10), Ufm1, Atg8 (Map1lc3b), Atg12,* and *Urm1*. No transcript for *Sumo2* or *Ubd* was found (TPM < 2) [25]. All the other genes were, however, significantly expressed, with *Atg8*, *Sumo3* and *Nedd8* displaying the highest levels of expression. For this set of genes, the TPM ranged from 70 to ⁓ 200 (Fig. 1B), a number that is approximately tenfold lower than that of Ub genes (Fig. 1A). *Atg8* was the major gene, and its expression was highly upregulated during development, with a number of transcripts showing expression increases by a factor of 3 between E11 and E17, suggesting activation of *Atg8*-dependent physiological processes, such as autophagy activation, at the end of cortical development (Fig. 1B). A recent proteomic analysis showed that the levels of conjugated and free NEDD8 (or ISG15) in the mouse brain were at least 60- and 20-fold lower than those of Ub [6], which is consistent with the profoundly lower gene expression levels of these genes, particularly *Isg15*, whose expression was negligible.

#### Ub-activating (E1) enzymes

Ubiquitination is a three-step enzymatic reaction. During the initial step, Ub is activated in an adenosine triphosphate-dependent manner by the Ub-activating (E1) enzyme before being transferred to a Ub-conjugating (E2) enzyme [2, 26]. Figure 1C shows the expression profile of the two genes *Uba1* and *Uba6* (*Ube1l2*) encoding mammalian Ub-activating E1 enzymes and seven genes encoding Ub-like proteins that activate E1 enzymes (*Uba2-3*, *Uba5*, *Uba7*, *Nae1*, and *Sae1*) [24]. The *Uba1* gene was by far the most prominently expressed E1 gene in the cerebral cortex (Fig. 1C). Its TPM values were ⁓ 200 on E11 and ⁓ 390 on E17, thus showing a nearly twofold increase in transcript abundance during embryonic development. Abundant expression of UBA1 may be a common feature of many cellular types, as the UBA1 protein is among the top 2% of the most highly expressed proteins in HeLa cells [18], reflecting the crucial requirement of this E1 enzyme in Ub-dependent cell processes. In the cerebral cortex, *Uba1* was the 597th and 343th most highly expressed gene on E11 and PN1, respectively, which confirms the relatively high abundance of *Uba1* transcripts. The *Uba1* gene product is abundant in the nucleus and cytoplasm [27], whereas the other mammalian E1 *Uba6* gene product is found only in the cytoplasm, which may ensure much more specific and restricted functions. *Uba6* was expressed at very low levels, with TPM values ranging from ⁓ 9 on E11 to ⁓ 4 on PN1. In the cerebral cortex, the ratios of *Uba1*:*Uba6* transcript abundance were > 20:1 and 90:1 on E11 and PN1, respectively. This differential expression was consistent with proteomic data showing that the relative abundance ratio of the UBA1 and UBA6 proteins is > 10:1 in HeLa cells [18], further suggesting a restricted function for UBA6 compared to that of UBA1. Collectively, the expression profile data of *Uba1* showed that it is the primary E1 gene in the cerebral cortex of mice. Due to its central role in Ub homeostasis, UBA1 is likely to regulate a wide range of neurobiological processes [28].

Far below the level of expression observed for *Uba1*, the expression of a set of six genes encoding Ub- and Ub-like proteins activating E1 enzymes (*Uba2-3*, *Uba5*, *Uba7*, *Nae1*, and *Sae1*) exhibited TPM values < 100. *Uba2* encodes an E1 enzyme specific for the Ub-like molecule SUMO. This *Uba2* gene product is thought to form heterodimers with SAE1 [24, 26]. Interestingly, the expression of both genes (*Uba2* and *Sae1*) was downregulated during development, with an abundance of transcripts reduced by nearly 50% between E11 and PN1 (Fig. 1C). Notably, no transcript for the Ub-like E1 gene *Atg7* was detected. Despite its low level of expression in the developing cortex, *Uba6* plays important roles in neuronal development, dendritic spine architecture, and mouse behaviour, and its deficiency is lethal [29]. Moreover, *Uba6* is required for neuronal viability in primary hippocampal neuronal cultures. Collectively, our data led to the identification of the highly regulated *Uba1* and *Uba2* genes as the major E1 and E1-like enzyme genes, respectively, expressed during cortical brain development.

#### Ub-conjugating (E2) enzymes

The analysis of the genes encoding Ub- and Ub-like protein-conjugating E2 enzymes [30, 31] is shown in Fig. 1D, E. Transcripts for *Ube2d4*, *Ube2e2*, *Cdc34b*, *Atg10* and *Ube2u* were not found. This observation reinforces the validity of our results since *Ube2u* transcripts were detected specifically in tissues of the urogenital tract [32]. All the other genes were expressed at significant levels (35 of 40 genes), particularly *Ube2m* (*Ubc12*), encoding a Nedd8-conjugating enzyme, with TPM values increasing from ⁓270 on E11 to ⁓320 on PN1 (Fig. 1D). The most important expressed genes encoding Ub-conjugating E2 enzymes were *Ube2c* and *Ube2r1* (*cdc34*) (Fig. 1D) and, to a lesser extent, *Ube2q1*, *Ube2ql1,* and *Ube2z* (*Use1*) (Fig. 1E). Expression of *Ube2c* was inhibited during embryonic development, with high levels of transcripts evident in the neurogenic period (E11–E13) and a marked decrease from E17 and later. Overall, TPM values of the *Ube2c* gene decreased by a factor of 25 between E11 (> 150 TPM) and PN1 (⁓ 7 TPM) (Fig. 1D). This decline represented the most downregulated genes in the Ub pathway during corticogenesis. The UBE2C protein is an exclusive partner of APC/C E3 ligases and controls cell cycle progression. Its mRNA and protein levels were low in quiescent cells but greatly increased and peaked during mitosis [31, 33]. *Ube2c* mRNA was thought to be barely detectable in tissues except under oncogenic conditions, with high levels in various cancers, such as brain and breast cancers [33]. The data presented in Fig. 1D show that high levels of *Ube2c* mRNA were found in nontumorous tissue under conditions not related to cancer onset or progression, similar to many developmental-specific genes whose re-expression is associated with carcinogenesis. Further studies are required to verify whether the UBE2C protein is a marker of neurogenesis in the brain.

*Ube2r1* was another prominently expressed Ub-conjugating E2 enzyme-encoding gene. Similar to that of *Ube2c*, the expression of *Ube2r1* was repressed, with an abundance of transcripts reduced by a factor of 4 between E11 and PN1 (from 126 to 34 TPM) (Fig. 1D). The E2 enzyme CDC34 (encoded by *Ube2r1*) is the primary E2 for cullin-RING E3 ligases (CRLs). Two members of the *Ube2q* gene family were expressed at moderate levels: *Ube2q1* and *Ube2ql1*. The expression of the *Ube2q1* gene was constant, showing no sign of developmental regulation. Western blot and immunohistochemical experiments showed the presence of UBE2Q1 proteins in the rat brain cortex, mainly in neurons [34]. UBE2Q1has been postulated to play an anti-apoptotic role, at least in pathological states such as traumatic brain injury [34]. The expression of *Ube2ql1* was not detected before E13, and it peaked in the E17-PN1 period, with TPM values increasing from ⁓8 to 85 TPM from E13 to E17. This increase represented an 11-fold increase in transcript abundance, making *Ube2ql1* the second most induced gene (in the Ub system) during embryonic development. In HeLa cells, UBE2QL1 exhibited a dual function: it is required for the efficient clearance of damaged lysosomes by lysophagy and maintains lysosome integrity [35].

The expression of minor genes (TPM values < 40) encoding Ub- and Ub-like proteins-conjugating E2 enzymes is reported in Additional file 1: Fig. S1A, B. The vast majority of these genes were expressed at constant levels, except *Ube2g2*, *Ube2s*, *Ube2t* and *Ube2l6*, which were downregulated. In particular, the expression *Ube2l6* was profoundly repressed, with TPM values decreasing from ⁓ 18 to ⁓ 2 (a ⁓ninefold reduction in transcript abundance) (Additional file 1: Fig. S1B), making it one of the most important downregulated genes observed in this study. Mutations in the *Ube2a* gene lead to neurodevelopmental disorders such as X-linked syndromic mental retardation. The precise roles *Ube2a* plays in brain formation are unknown. In the rodent brain, the *Ube2a* gene was expressed at low levels, with few transcripts evident throughout cortical development (Additional file 1: Fig. S1A). It has been proposed that some of the cellular effects of UBE2A involve the E3 Ub ligase Parkin. This might be the case in adults, but this enzyme was not expressed during embryonic development of the cerebral cortex, suggesting the involvement of other E3 partners (see below, “RING E3 Ub ligases”). UBE2A exerts some of its actions via the E3 Ub ligases RAD18 and RNF20. The UBE2A/RAD18 complex is at least partially responsible for the pathogenesis of mental retardation (in association with the proliferating cell nuclear antigen, PCNA [12]). The *rad18* gene was expressed uniquely on E11 and E13, but the abundance of transcripts was very low (< 6 TPM), suggesting that its presence is required for brain development exclusively during neurogenesis.

This transcriptomic analysis provides a detailed overview of the expression patterns of E2 genes that are central players in the Ub pathway. Overall, these genes were expressed at low levels during embryonic development of the mouse cerebral cortex. As in HeLa cells or Swiss 3T3 cells, *Ube2m* (encoding UBE2M/UBC12 and involved in neddylation) was the prominent E2. However, the pattern of expression of the Ub- and Ub-like protein-conjugating E2 genes in the cerebral cortex did not completely overlap with that reported in cell lines. For instance, UBE2I/UBC9 (encoding SUMO) and UBE2N were abundant E2 proteins in cell lines [18]. Together with UBE2V1, UBE2N represents > 50% of the Ub-dedicated E2 enzymes in HeLa cells, and it is associated with UBE2V2 in Swiss 3T3 cells [18]. UBE2L3 is another abundant E2 that is expressed at levels twofold higher that all HECT and RBR E3 ligase genes in HeLa cells combined [18]. This expression pattern is profoundly different than that of the cerebral cortex, where the *Ube2l3* and *Ube2n* genes were expressed at moderate levels (TPM values of 20–30) (Additional file 1: Fig. S1). The E2 enzyme UBE2L3 works in concert with the E3 ligase Ube3a (also known as E6-associated protein or E6-AP) [36]. Mutations and genetic defects in the *Ube3a* gene are associated with the Angelman syndrome, a neurodevelopmental disease [37]. The profile of E2 enzyme expression in nontumorous tissue is likely to differ from that in immortalized cells. Our data clearly illustrate that the pattern of E2 gene expression was temporally regulated. This pattern may, however, differ from one brain area to another.

#### Ub ligases (E3)

Ub ligase (E3) enzymes exert two crucial functions: they target a specific type of ubiquitinated substrate and enable the final transfer of Ub (to the substrate) [38]. In this study, E3 Ub ligases are grouped into 3 families according to [39]: the HECT (homologous to the E6-AP carboxyl terminus), RBR (RING-in-between-RING), and RING (really interesting new gene) E3 families. Depending on the E3 ligase, the transfer of Ub from the E2 enzyme to the target substrate can occur directly or via a 2 step process. For instance, RING E3 ligases enable a direct transfer of Ub from E2 to the target whereas the ubiquitination involving HECT develops via a 2 step process with Ub carried by the E2 enzyme binds first to a HECT domain before being transferred to the target protein [38, 39]. Although RBR E3 ligases have two RING domains and could be categorised as a sub-class of RING-type ligases, they are described as RING-HECT hybrids catalysing ubiquitination not directly like RING-type ligases but via a two-step reaction like HECT-type ligases during which Ub is transferred to the RING2 domain and then to the target [38–41].

**Fig. 2:**
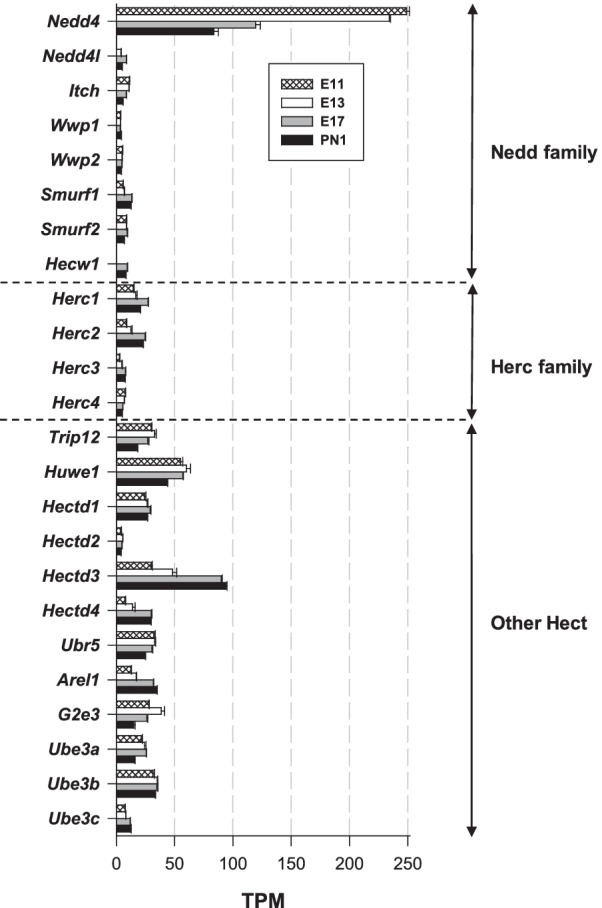
Expression of the genes encoding HECT Ub E3 ligases. The figure shows the abundance of three groups of transcripts: Nedd, Herc and other HECT

*HECT Ub ligases* Genes were subdivided into 3 groups: Nedd, Herc and other HECT ligases [42]. Transcripts of twenty-four HECT genes were found (Fig. 2), with four genes (*Ube2cbp*, *Hace1*, *Herc6*, *Hecw2*) below the detection limit. The group of HECT E3 Ub ligases was dominated by the high abundance of *Nedd4* transcripts. The TPM values decreased from 249 (on E11) to 84 (on PN1), reflecting a ⁓ threefold reduction in transcript abundance throughout corticogenesis. *Nedd4* was the most highly expressed HECT E3 gene during neurogenesis (E11–E13) and the second most highly expressed gene at the end of corticogenesis (E17-PN1), after *Hectd3*, the other prominent HECT gene. Our data were in line with the first study reporting the isolation of both a set of Nedd4 cDNA clones and the corresponding mRNA expression [43]. This later study also showed a gradual mRNA decrease during embryonic development in the brain. *Nedd4l*, which is closely related to *Nedd4*, the founding and most ancient member of the Nedd4 family, was expressed on E13 and onwards at a very low level (TPM values < 9). These data illustrate the temporal regulation of these E3 Ub ligases, which play essential roles in neuronal cell fate determination and survival, neurite outgrowth, axon guidance and branching [44].

Compared to HECT, E3 Ub ligases in the Herc group were expressed at much lower levels, with only *Herc1* and *Herc2* showing a TPM reaching a maximum of 23, on E17 (Fig. 2). In the third category (i.e., the HECT E3 Ub ligases that differ from Nedd and Herc), *Hectd3* was the predominant gene, with highly regulated expression during corticogenesis, with TPM values increasing by a factor of 3 from E11 to PN1 (from ⁓ 30 to ⁓ 94 TPM) (Fig. 2). *Huwe1* was the third most highly expressed gene of this subfamily of HECT E3 ligases, with TPM values of 43–60. Interestingly, knocking down HUWE1 expression in cortical tissue with a siRNA resulted in an increase in the fraction of proliferating cells in the developing brain and blockade of neuronal differentiation [45]. These results show that HUWE1 controls neural differentiation and proliferation. All the other genes in this subgroup were expressed at low levels, with TPM values < 30 (Fig. 2). The HECT E3 Ub ligase UBE3A has been well characterized because mutations in the *Ube3a* gene cause Angelman syndrome, a neurodevelopmental disease [37]. However, *Ube3a* was expressed at low levels throughout the cortex (TPM values of 16–25, Fig. 2). Notably, the highest abundance of HECT Ub ligase gene transcripts was noted at the peak of neurogenesis (E13) and then declined.

*RING E3 Ub ligases* RING E3 ligases constitute the largest family of E3 Ub ligases [8] [38]. For instance, a previous analysis of the mouse genome identified 398 putative E3 enzymes [46]. In the following sections, RING E3 Ub ligase genes are classified into three main subgroups: (1) single subunit, (2) multiple subunit RING E3 and (3) U-box RING E3 ligases.

**Fig. 3:**
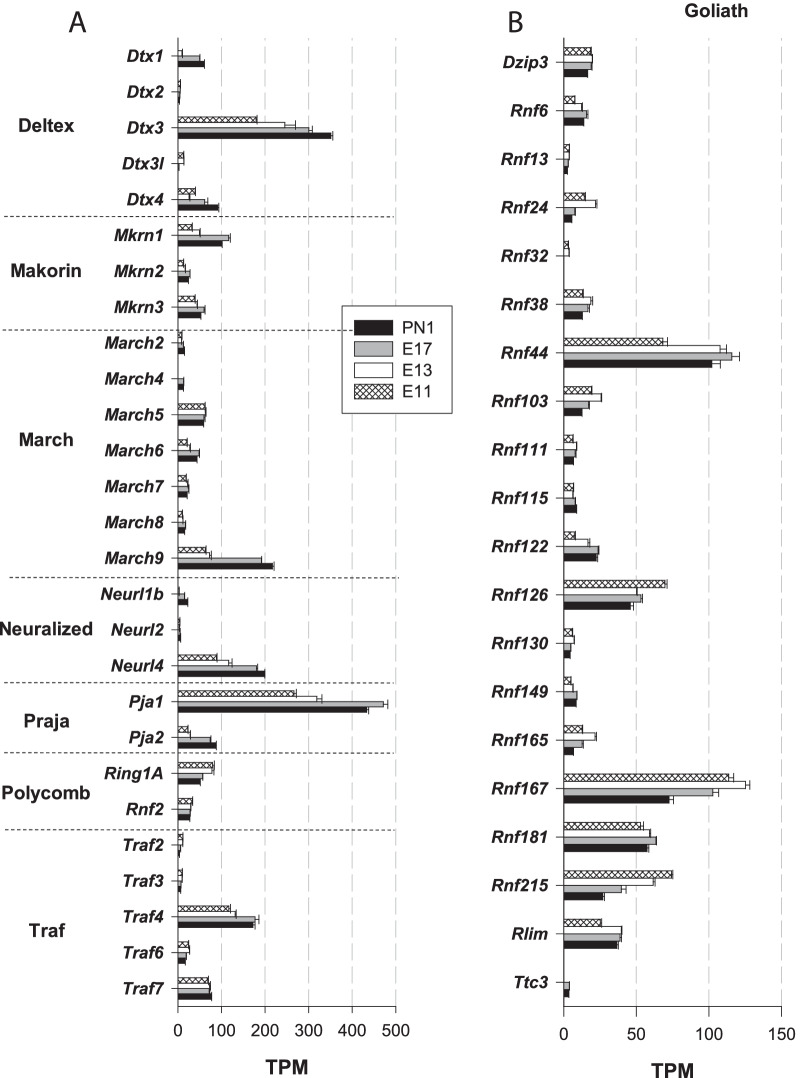
Expression of the major genes encoding a single subunit of RING E3 ligases. This figure shows the most highly expressed RING E3 ligase gene families: *Deltex, Makorin, March, Neuralized, Praja, Polycomb, Traf* (**A**) and *Goliath* (**B**)

**Fig. 4:**
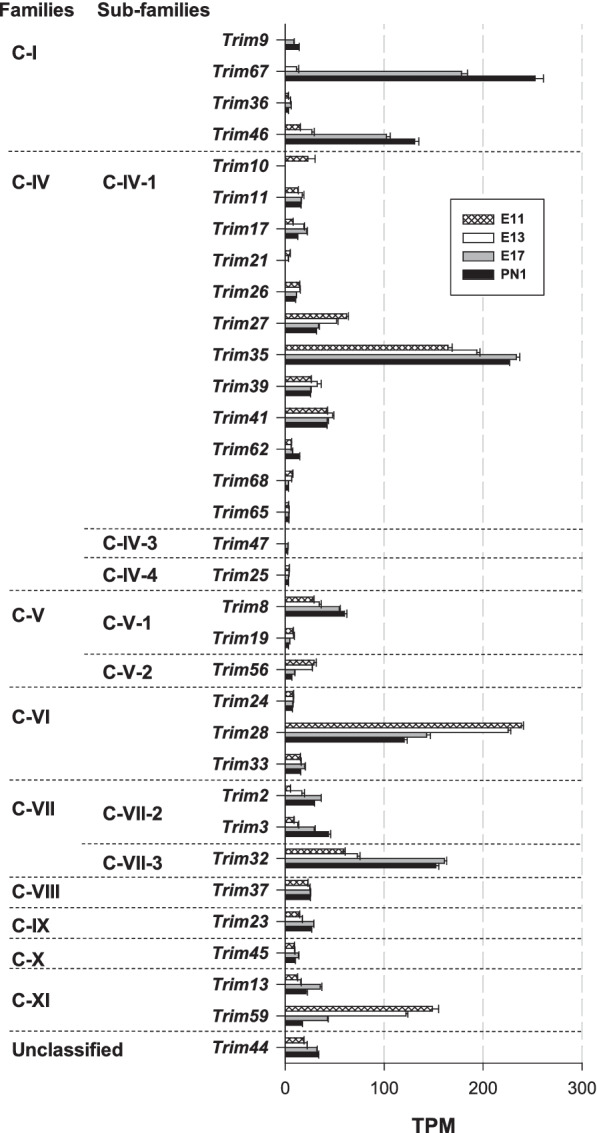
Expression of the *Trim* genes encoding Ub E3 ligases of the RBR type. In this analysis, several families and subfamilies of *Trim* genes (C-I to C-XI) and *Trim4*, encoding a protein lacking a RING-finger domain, were analysed

**Fig. 5:**
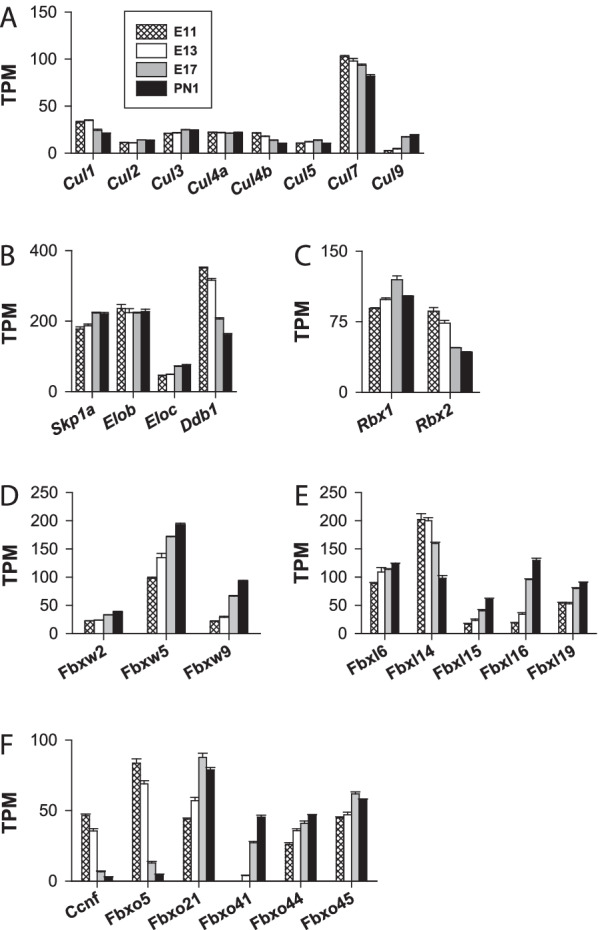
Expression of genes encoding cullin scalfolds, adaptor proteins and RING finger proteins. Transcripts of cullin (*Cul*) (**A**), adaptor proteins (**B**) and RINGER proteins (**C**) genes are shown. **D**, **E** and **F** show the pattern of expression of the major *Fbxw*, *Fbxl* and *Fbxo* genes. They all had TPM values ≥ 40. The minor *Fbxw*, *Fbxl* and *Fbxo* genes are shown in Additional file 1: Fig. S2A–C

**Fig. 6:**
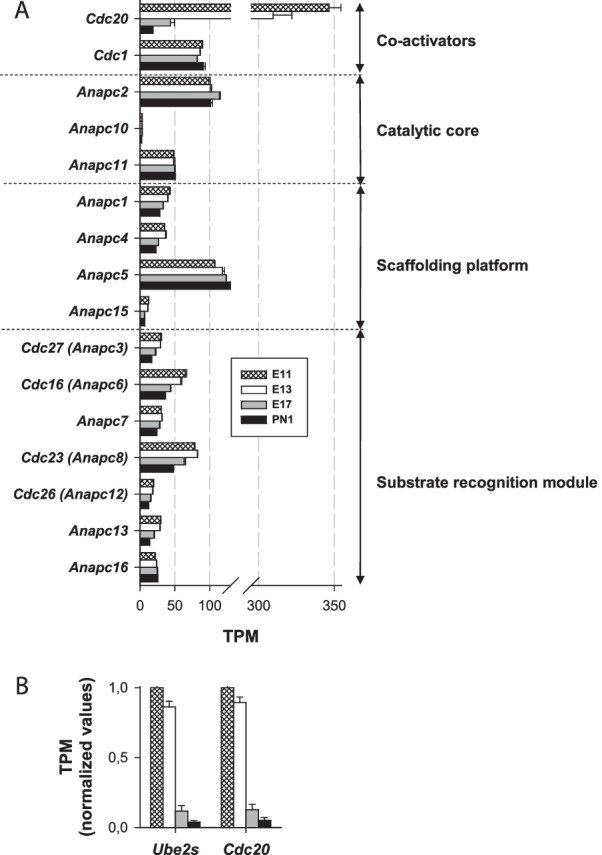
Expression of E3 Ub ligase anaphase-promoting complex/cyclosome (APC/C) and coactivator-encoding genes. This figure shows the expression of fourteen APC-encoding genes (*Anapc*) and two coactivator-encoding genes (*Cdc20* and *Cdh1*) (**A**). **B** shows the normalized expression of *Cdc20* and *Ube2c*, a gene encoding an E2 Ub-conjugating enzyme working in conjunction with the APC/C E3 Ub ligase

**Fig. 7:**
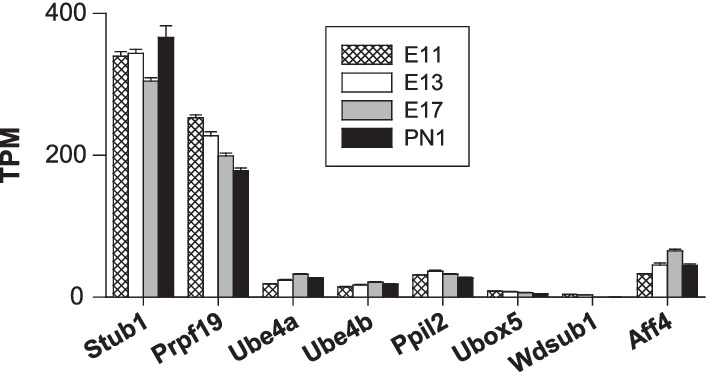
Expression of U-box E3 Ub ligase genes. The genes analysed were *Stub1 (Chip), Prpf19 (Prp19), Ube4a (Ufd2b), Ube4b (Ufd2a), Ppil2 (Cyc4), Ubox5 (Uip5), Wdsub1, Act1 (Traf3ip2)* and *Aff4*. However, *Act1* was not expressed

A. Single subunit RING E3:
  A list compiled by [47] was used for the analysis of the major E3 ligase subgroups: *Cbl, Deltex, Goliath, IAP, Listerin, Makorin, MARCH, Neuralized, Pellino, Pex, Polycomb, Praja, RBR, Siah, Traf, Trim* and *Ubr*. Notable heterogeneity in expression levels was observed among these genes. The most highly expressed subgroup genes included *Deltex, Goliath, Makorin, March, Neuralized, Praja, Polycomb, Traf* (Fig. 3) and *Trim* genes (Fig. 4). The expression levels of the other minor gene groups (*Cbl, IAP, Listerin, Pellino, Pex, Siah,* and *Ubr*) are presented in Table 1. The TPM values of all the genes in this set were < 37, except the *Ubr7* gene, for which the TPM value was ⁓ 60 on E11 and E13.
  *Deltex E3 Ub ligases* *Dtx3* and *Dtx4* were the major *deltex* genes, with TPM values increasing from 179 to 351 (*Dtx3*) and from 40 to 91 (*Dtx4*) (Fig. 3A), revealing strong positive regulation during corticogenesis. However, the most highly regulated gene in this group was *Dtx1*: no transcripts were detected on E11, but it was clearly strongly induced later, with TPM values increasing from 9 (on E13) to 60 (on PN1), a nearly sevenfold increase (Fig. 3A). Deltex E3 has been principally studied in the context of tumorigenesis and tumour cell invasion [48], but very little is known about the roles played by Deltex E3 Ub ligases in the developing or adult brain in mammals. The marked enhancement of *deltex* gene expression supports the notion suggesting key roles in neuronal growth and differentiation in mammals.
  *Goliath E3 Ub ligases* Twenty-nine orthologous genes of the Drosophila Goliath E3 Ub ligases were identified in mice (https://flybase.org/reports/FBgg0000104.html). However, nine of these genes were not expressed (*Rnf43*, *Rnf128* (*Grail*), *Rnf133, Rnf148, Rnf150, Znrf3, Znrf4, Zswim2* and *4930595M18Rik*). In the expressed gene subgroup, *Rnf44* (68–115 TPM), *Rnf167* (72–125 TPM), and *Rnf126* (46–70 TPM) were the major genes (Fig. 3B). Notably, *Rnf215* was the most highly regulated gene, with the abundance of its transcripts decreasing from 74 to 27 TPM from E11 to PN1, a 2.7-fold decrease during corticogenesis. The contribution of RNF44 to brain formation and functions is unknown. The E3 ligase RNF167 plays important roles in neuronal cells. Although principally found in lysosomes, a fraction of RNF167 is present at the cell surface, where it participates in the ubiquitination of AMPA receptors. Ubiquitination modulates the number of AMPA receptors at the cell surface as well as synaptic currents. Therefore, RNF167 is an important physiological modulator of glutamatergic neurotransmission [49]. This RNF167-dependent ubiquitination of AMPA receptors was recently shown to be mediated by the E2 enzymes Ube2D1 and Ube2N [50]. RNF126, another prominent factor in this subgroup, has been shown to be involved in Friedreich ataxia, a severe genetic neurodegenerative disease characterized by reduced expression of the essential mitochondrial protein frataxin. The E3 Ub ligase RNF126 specifically mediates frataxin ubiquitination, which induces its degradation [51]. Our results point towards a role played by Goliath E3 Ub ligases in neuronal function from early embryonic stages.
  *Makorin E3 Ub ligases* All three makorin genes (*Mkrn1-3*) were expressed in the immature cerebral cortex (Fig. 3A). *Mkrn1* was the predominant gene, with TPM values increasing from 32 (on E11) to 116 (on E17). Consistent with our findings, *Mkrn1* had been originally identified as a highly expressed gene during mouse embryonic development, with a high level of mRNA expression in the developing brain [52]. Low levels of Mkrn1 proteins were found in the brain despite its relatively high mRNA abundance due to the autoubiquitination properties of this E3 Ub ligase, which induces its own proteasomal degradation [53]. Experiments performed with Xenopus embryos showed that Mkrn2 proteins inhibit neurogenesis by acting downstream of phosphatidylinositol 3-kinase (PI3K) and Akt [54]. Thus, Mkrn proteins clearly play major roles in the developing nervous system.
  *MARCH E3 Ub ligases* The family of proteins of the membrane-associated RING-CH (MARCH) comprises eleven E3 Ub ligases (MARCH-1 to -11) [55]. Four *March* genes of the eleven analysed in this study were not expressed: *March-1*, *-3, -10* and *-11*. The major gene in the group was *March 9*. Its TPM value was approximately 60 on E13, which was profoundly increased on E17 (192 TPM) and PN1 (217 TPM), corresponding to a > 3.5-fold increase (Fig. 3A). On E17 and PN1, *March 9* transcripts represented more than 50% of all *March* transcripts. In dendritic cells, MARCH-9 proteins localize to the trans-Golgi network (TGN) and controls a TGN‐to‐endosome transport step [56]. In previous studies, MARCH-9 expression had been mainly found in immune cells and organs such as the lung, lymph nodes, and the spleen, not neuronal cells [57]. Our data, however, indicate that MARCH-9 was highly expressed at the end of neurogenesis. Clearly, additional work is needed to delineate the neuronal functions of MARCH-9 proteins in the brain. Although expressed at a considerably lower level, the other major *March* gene was *March-5*, a mitochondrial-associated E3 Ub ligase. Its TPM values were 58–62, and showed no sign of development regulation. Of note, transcripts of *March 4* were detected on E17 and onwards with TPM values of ⁓ 12 (Fig. 3A). MARCH-4, a Golgi-associated E3 Ub ligase, was the only member of the MARCH family previously known to be expressed in the brain [57], but the TPM values were low (less than 13 TPM) (Fig. 3A). Our transcriptomic analysis revealed a large repertoire of factors, with seven *March* genes expressed throughout corticogenesis and both high and highly regulated expression of *March-9* (Fig. 3A).
  *Neuralized E3 Ub ligases* Transcripts of three neuralized genes were measured: *Neurl1b*, *Neurl2* and *Neurl4*. The expression of the major gene *Neurl4* was enhanced, with TPM values increasing from ⁓ 90 to ⁓ 200 (Fig. 3A). The protein NEURL4, **found** in the developing rodent cerebellum [58], is a p53-interacting protein that when overexpressed inhibits cellular growth [59]. Furthermore, previous experiments preformed with NEURL4-knockdown animals showed a reduced number of presynaptic boutons, indicating that NEURL4 regulates synapse development in the brain [58], consistent with its upregulated expression during corticogenesis.
  *Praja E3 Ub ligases* The expression of the two Praja genes *Pja1* and *Pja2* was positively regulated during corticogenesis. The abundance of *Pja1* and *Pja2* transcripts increased by factors of 1.6 and 3.8, respectively (Fig. 3A). With expression less regulated than that of *Pja2*, the *Pja1* gene was the predominant *Praja* gene and one of the most highly expressed genes in the Ub system. Its TPM values were on the order of 270 (on E11) and ⁓ 430 (PN1), peaking on E17 (⁓ 480) (Fig. 3A). On average, *Pja1* transcripts were 11- and 5–sixfold more abundant than *Pja2* transcripts on E11-E13 and E17-PN1, respectively. The human and mouse *Pja1* genes are highly expressed in the brain, particularly in the cerebral cortex [60]. Northern blot experiments showed *Pja1* mRNA in the immature brain on E11.5. Suppression of *Pja1* expression led to a high apoptosis rate, indicating that the protein exerts a prosurvival anti-apoptotic effect. In line with the function of the encoded protein in cell survival, *Pja1* mRNA has been previously found to be overexpressed in twenty-nine cancer types, with particularly high expression in gliomas [60]. Altogether, these results support the idea that Praja1 proteins play important roles in brain development and regulation of cell apoptosis.
  *Polycomb complexes* Polycomb-containing complexes possess E3 Ub ligase activity due to its RING1A (*Ring1*) or RING1B (*Rnf2*) member. The abundance of *Rnf2* transcripts did not vary during corticogenesis, whereas a reduction in *Ring1* transcripts was observed between E11 and E17, with TPM values decreasing from 80 to 50 (Fig. 3A).
  *Traf E3 Ub ligases* Transcripts of four *Traf* genes were identified, but their levels varied, with the predominant being *Traf4* with TPM values of 120–180 (Fig. 3A). In contrast to the other *Traf* members, *Traf4* expression was increased during corticogenesis. TRAF4 proteins are essential for neural crest development and neural folding in Xenopus [61]. In mice, TRAF4 deficiency can induce defects in neural tube closure [62]. This protein also participates in the control of myelination [62]. However, transcripts of two *Traf* genes (*Traf1* and *Traf5*) were not detected in the present study.
  *TRIM E3 Ub ligases* Proteins of the tripartite motif (TRIM) family are engaged in multiple cellular processing through their E3 Ub ligase activity. Absent in yeast, TRIM proteins are required for activation of mammalian autophagy and critical for the regulation of innate immunity [41]. Several families and subfamilies of TRIM proteins have been identified (C-I to C-XI), in addition to a group of unclassified TRIM proteins lacking a RING-finger domain [41]. More than eighty genes were analysed in this study. Taken together, the data revealed that transcripts of thirty-two genes encoding RING-finger domain-containing TRIM and only one TRIM without a RING-finger domain were found (Fig. 4). Six *Trim* genes were very highly expressed: *Trim28*, *Trim32*, *Trim35*, *Trim46*, *Trim59* and *Trim67*. The latter was both the most highly expressed *Trim* gene and one of the most highly upregulated genes analysed in the present study. No transcripts were detected before E13, and the TPM values increased from 12 (on E13) to 253 (on PN1). Overall, the transcript abundance increased by a factor of 21 during embryonic development. The most significant increase was noted between E13 and E17, indicating that TRIM67 is a dispensable ligase during neurogenesis but is crucial for postmitotic cell functions and the maturation of the cerebral cortex (Fig. 4). These data are in line with a previous report showing that TRIM67 proteins are highly expressed in the developing and mature brain but not found in nonneuronal tissues [63]. The TRIM67 protein expression peaked late in the embryonic and perinatal stages, indicating that it is involved in neuronal development after the proliferative period. Deletion of the *Trim67* gene causes malformations in several brain regions associated with cognitive and behavioural impairments [63]. The molecular role played by TRIM67 in brain development as well as the nature of its substrates are, however, unknown.
  *Trim35* expression was not regulated to the same extent as that of *Trim67,* but *Trim35* was nevertheless expressed at all ages. *Trim35* TPM values increased from ⁓ 170 to 230 from E11 to PN1, reflecting a 35% augmentation in transcript abundance (Fig. 4). The third most prominent gene in this family was *Trim28*. Its expression was downregulated, with TPM values decreasing from ⁓ 240 to 120 between E11 and PN1. The repression of *Trim28* expression was evident after E13, indicating that TRIM28 (KAP1 or TIF1b) exerts important effects during the proliferative period. TRIM28 is an epigenetic corepressor protein highly expressed both in the developing and adult brain [64]. Its absence in mice is embryonically lethal (on approximately E5.5). TRIM28 has been proposed to be a SUMO E3 ligase [65]. In murine and human brains, TRIM28 functions as a transcriptional regulator of neurodevelopmental gene programmes important for brain development [64].
  The other main *Trim* genes were found to be *Trim32*, *Trim46*, and *Trim59*. The expression of *Trim32* and *Trim46* was upregulated: the abundance of their transcripts increased markedly after E13. For instance, the TPM values increased by a factor of 2.6 and 8.7 for *Trim32* and *Trim46*, respectively, between E11 and PN1 (Fig. 4). *Trim46* was one of the most induced genes (an ⁓ ninefold increase). Accumulation of TRIM32 proteins into neural cells favours their commitment to the neuronal lineage [66]. Following its translocation to the nucleus, TRIM32 targets c‐Myc for proteasomal degradation, which initiates neuronal differentiation [66]. TPM values for *Trim59*, another highly regulated gene, decreased by a factor of ⁓ 9 (from 149 to 17 TPM) (Fig. 4). These changes in transcript abundance were primarily identified after the peak of neurogenesis (on E13). The mRNA levels were much higher during the proliferative periods of corticogenesis. TRIM59 proteins are abundantly expressed in certain organs, such as the spleen, stomach and ovary, but they are also found at lower levels in the brain, lung, kidney, muscle and intestine [67]. Again, it is interesting to note the high expression level of factors known to regulate carcinogenesis. For instance, TRIM28, TRIM32, and TRIM59, which have been found to be aberrantly overexpressed in certain cancers, were highly abundant in this study. Specifically, TRIM28 has been associated with proteins of the melanoma-associated antigen (MAGE) family and favours the progression of carcinogenesis via suppression of autophagy [68]. Notably, many E3 Ub ligases, such as MARCH and TRIM proteins, known for the roles they play in immune responses, were highly expressed in the developing cerebral cortex.
B. Multisubunit RING E3 ligases:
  Three families of multimeric RING E3 ligases were considered in the present report: (1) cullin RING ligases, (2) the APC/C E3 ligase, and (3) the Fanconi anaemia complex.
  1. Cullin RING ligases (CRLs)
    Cullin RING ligases (CRLs) represent the largest family of E3 Ub ligases. They are complex molecular entities with several independent subunits. CRLs (CRL1-9) comprise a cullin (Cul) scaffold associated with a RING-box protein and an adaptor protein. They also require a substrate recognition element that is an interchangeable subunit that indicates the target protein to be ubiquitinated. CRL3 is a notable exception, because the same molecular entity (Broad complex, Tramtrack, Bric-a-brac, the BTB domain) is both an adaptor and substrate receptor [39]. Several cullins, RING-box proteins, adaptor proteins, and hundreds of substrate recognition proteins contribute to the generation of a wide range of combinations giving rise to a multitude of functionally distinct CRLs [69]. Table 2 presents an overview of the multisubunit structure of CRLs and their modularity.
    B.1.1 Cullin scaffold proteins
      Transcripts of nine cullin genes (*Cul1-3*, *4a*, *4b*, 5*, 7*, and *Cul9* or *Parc*) were detected, with *Cul7* being the predominant member of this group. *Cul7* TPM values slightly decreased from 103 to 82 from E11 to PN1 (Fig. 5A). The CUL7 protein, present only in chordates, participates in the control of embryonic development. CUL7-knockout mice displayed neonatal lethality, and mutations in the human *Cul7* gene were linked to the growth retardation disorder 3-M syndrome [69]. The expression level of the human *Cul7* gene is increased in glioblastoma tissues compared to normal brain tissues. Furthermore, *Cul7* facilitates the proliferation, invasion and migration of glioma cells by activating the NF-κB pathway [70]. These effects are consistent with the current view that CUL7 is an oncogene [71]. *Cul7* expression is elevated in healthy (nontumorigenic) brain tissue. The neuronal functions of the scaffold protein CUL7 are not clear but it has been found to be highly abundant in the developing rat brain [72, 73]. Only two F-box proteins are known to interact with CUL7: FBXW8 and FBXW11 [71]; the gene expression of these proteins showed an opposite pattern: the abundance of *Fbxw8* transcripts decreased from 16 to 10 TPM, whereas the abundance of *Fbxw11* transcripts increased from 12 to 19 TPM from E11 to PN1. CUL7 is, together with the F-box protein FBXW8, associated with the Golgi apparatus in neuronal cells and is required for the growth of dendrites (but not axons) in neurons in the mammalian brain [72]. CUL7 is also found at synaptic sites, controlling the degradation of Eag1, a potassium channel in the plasma membrane that participates in the regulation of membrane excitability [73]. Due to its high expression level and synaptic localization, CUL7 may be an important modulator of neuronal excitability in the brain. Within the CRL7 E3 ligase complex, the scaffold protein CUL7 is associated with the adaptor protein Skp1 and the RING finger protein ROC1, which contains a E2 enzyme-binding domain. The expression of the *Skp1* and *Rbx1* genes is discussed below. The other *Cul* genes showed low TPM values (approximately 10–20 TPM). The expression of the minor *Cul9* gene was developmentally regulated: its transcript abundance was increased by a factor of ⁓ eight between E11 and PN1 (from ⁓ 2 to 19 TPM) (Fig. 5A). It was one of the most upregulated genes analysed in this study.
    B.1.2 Adaptor proteins
      Adaptor proteins are attached to the cullin scaffold. The following four genes were identified in this study: *Skp1* (*Skp1a*), *Elob* (*elongin B*), *Eloc* (*elongin C*) and *Ddb1*. Three of these genes were highly expressed with TPM values ≥ 180: *Skp1a*, *Elob*, and *Ddb1* (Fig. 5B). They displayed distinct patterns of expression: *Elob* was expressed at a constant level, whereas *Ddb1* expression was negatively regulated (Fig. 5B). Transcripts of *Ddb1*, the major gene in this subgroup, were reduced drastically during embryonic development, with the TPM value decreasing from 351 to 162 (Fig. 5B).
    B.1.3 RING finger proteins
      The RING finger E3 ligases function as docking sites for E2 enzymes. The TPM values of the *Rbx1* and *Rbx2* (*Rnf7*) genes were nearly identical on E11 (89 and 86, respectively) (Fig. 5C). These genes displayed differing expression patterns: the abundance of *Rbx1* transcripts increased (with a peak on E17 with 109 TPM), but that of *Rbx2* was decreased (with 43 TPM on PN1). Overall, *Rbx1* was the predominant RING finger protein-encoding gene in the cerebral cortex during development (Fig. 5C).
    B.1.4 Substrate receptors
      In this chapter, we have analysed the expression of genes encoding F-box proteins. They are important components of the Skp1-Cullin 1-F-box complex (SCF E3 Ub ligases). F-box proteins play critical roles as substrate receptors and have been classified into three groups: FBXW, FBXL and FBXO F-box proteins [74]. The analysis of the genes (*Fbxw*, *Fbxl* and *Fbxo*) followed this classification. The most highly expressed genes (with TPM values ≥ 40) are shown in Fig. 5D–F. The expression of the minor F-box genes (TPM values < 40) is presented in Additional file 1: Fig. S2.
    *Fbxw* genes: The major genes of this subgroup were *Fbxw2*, *Fbxw5* and *Fbxw9* (Fig. 5D). They were all upregulated during corticogenesis, particularly *Fbxw9* with TPM values increasing ⁓ fourfold from 22 (on E11) to 93 TPM (on PN1). *Fbxw5* was the most highly expressed *Fbxw* gene. The TPM values increased from 98 to 193, a ⁓ twofold increase in transcript abundance (Fig. 5D). This is another example of a member of the Ub system known for its contribution in tumorigenesis [75] that is highly expressed in a nontumorous brain tissue. However, the roles of FBXW5 proteins in the brain are unknown. The minor *Fbxw* genes displayed TPM values ranging from 2 to 20. Similar to major *Fbxw* genes, the expression of the minor *Fbxw* genes was positively regulated during corticogenesis except *Fbxw8* (Additional file 1: Fig. S2A). Of note, two *Fbxw* genes were not expressed (*Fbxw10* and *Fbxw12*).
    *Fbxl* genes: The major *Fbxl* genes were *Fbxl6*, *Fbxl16, Fbxl19* associated with the predominant *Fbxl14* gene (Fig. 5E). This gene had elevated TPM values that decreased during corticogenesis (from 202 to 98 TPM). The proteins FBXL14 has been reported to associate with Hes1 (hairy and enhancer of split 1), a repressor of proneural genes. Furthermore, the loss or overexpression of FBXL14, respectively, stabilizes Hes1 or decreases its protein levels [76]. In stem cells, FBXL14 controls the proteasomal degradation of Hes1, which favours neuronal differentiation [76]. The temporal pattern of expression of the *Hes1* gene is reported in Fig. 9B. Both genes, *Fbxl14* and *Hes1*, were downregulated during corticogenesis. The other major *Fbxl* genes had however a different pattern of expression with an abundance of transcripts increasing significantly during embryogenesis. The expression of the *Fbxl16* gene was strongly induced with TPM values increasing from 19 (on E13) to 130 (on PN1), a nearly sevenfold increase (Fig. 5E). The temporal pattern of expression of the minor *Fbxl* genes is shown in Additional file 1: Fig. S2B. No transcripts of the following six *Fbxl* genes (*Fbxl4, Fbxl7, Fbxl8, Fbxl13, Fbxl17, and Fbxl21*) were detected.
    *Fbxo* genes: Amongst the six genes of this group that had TPM values > 40: *Ccnf* (*Fbxo1*), *Fbxo5, 21, 41, 44,* and *45*, two *Fbxo* genes predominated: *Fbxo5* and *Fbxo21* (Fig. 5F). They had however differing expression patterns: the expression of *Fbxo5* was strongly reduced (TPM decreasing from 84 to 5, a 17-fold reduction in transcript abundance) while the expression of *Fbxo21* was induced during corticogenesis (TPM values increasing from 44 to 79, a 1.8-fold increase) (Fig. 5F). Although expressed at lower levels (TPM values ranging from 46 to 3), *Ccnf* was also strongly downregulated. The abundance of *Ccnf* transcripts decreased nearly 15-fold from E11 to PN1 (Fig. 5F). *Ccnf* and *Fbxo5* seemed to play critical roles at the onset of neurogenesis. Fbxo5 proteins have been shown to control cell proliferation [77]. The expression of *Fbxo41* was strongly induced: no transcripts were detected at E11 but the abundance of transcripts increased from 4 TPM (on E13) to 45 TPM (on PN1) (Fig. 5F), a 11-fold increase. Fbxo45 proteins are found exclusively in the brain [78, 79] and Fbxo45 mRNA is detected as early as E12 [79]. The loss of Fbxo45 is postnatally lethal and is associated with an abnormal embryonic neural development [78]. Fbxo45 proteins play important roles in the brain by regulating neurotransmission [79]. The *Fbxo45* gene expression was significantly enhanced at the end of neurogenesis (Fig. 5F). It is however important to note that Fbxo45 fails to associate with Cul1 and does not form an SCF complex but associates with a RING finger-type Ub ligase [80]. The expression of the minor *Fbxo* genes is shown in Additional file 1: Fig. S2C. The number of transcripts of seven *Fbxo* genes were below the detection threshold (*Fbxo15, Fbxo16, Fbxo24, Fbxo36, Fbxo39, Fbxo40, and Fbxo23*).
  B.2. The anaphase-promoting complex/cyclosome (APC/C) E3 ligase
    The E3 Ub ligase anaphase-promoting complex/cyclosome (APC/C) is well known for its control of the cell cycle because it regulates mitotic progression and exit. It is highly abundant in postmitotic neurons, where it plays a role in dendrite and axon arborization and in synaptogenesis [9]. The APC/C E3 ligase is a multi-subunit complex displaying a similar structure to the Skp1/Cul1/F-box protein Ub ligases. Both are composed of three fixed subunits (a catalytic RING protein, a scaffold protein and an adaptor protein) and another component conferring substrate specificity (an F-box protein for SCF, and Cdh1 or Cdc20 for APC/C) [81].
    The three sub-complexes of the APC/C ligase consist in a catalytic core (with APC2, APC10, or APC11), a scaffolding platform (APC1, APC4, APC5, or APC15), and a substrate recognition module (or tetratricopeptide repeat lobe, TPR) (consisting of APC3, APC6, APC7, APC8, APC12, APC13, of APC16). In addition, CDC20 and CDH1 are coactivators (also considered to be substrate receptors) essential for the activity of an APC/C ligase [82, 83]. The expression of fourteen APC-encoding genes (*Anapc*) and two coactivator-encoding genes (*Cdc20* and *Cdh1*) was analysed.
    *Anapc2* (100–115 TPM) and *Anapc11* (⁓ 49 TPM) were the major genes of the catalytic core. Transcripts of the other component (*Anapc10*) were near the detection level (2–3 TPM) (Fig. 6A). With TPM values ranging from 128 to 107 from E11 to PN1, *Anapc5* was the predominant gene in the scaffolding platform. *Anapc1* and *Anapc4* presented comparable low levels of expression (TPM values of 20–40), whereas *Anapc15* was expressed at even lower levels (TPM values of 6–12) (Fig. 6A). *Anapc6* (*Cdc16*) and *Anapc8* (*Cdc23*) were the most highly expressed genes in the substrate recognition module (Fig. 6A). Taken together, the transcripts of this subgroup had low or moderate abundance, with TPM values ranging from 12 to 64 TPM. As shown in Fig. 6A, the expression of genes in the APC/C subgroup displayed nearly constant transcript numbers throughout cortical formation, suggesting their basic functions in cell physiology. Notably, the extremely elevated expression of the *Cdc20* gene at early stages of cortical development was followed by a sharp decrease on E17. Its TPM values declined from ⁓ 350–310 on E11–E13 to 44–18 on E17-PN1. Overall, the abundance of *Cdc20* transcripts was reduced by a factor ⁓ 20, showing that this gene exhibited a marked temporal pattern of expression. The high abundance of *Cdc20* transcripts corresponded to periods of cell production (E11-E13), strongly suggesting a role for *Cdc20* in cell proliferation. The expression of the other coactivator-encoding gene *Cdh1* (*Fzr1*) was not developmentally regulated. Constant levels of *Cdh1* transcripts were found during embryonic development (TPM values of 89–90). Chd1 proteins are required for neurogenesis in vivo [84]. It seemed, however, that the regulation of the coactivator gene *Cdc20* expression was a central determinant affecting the functionality of the APC/C E3 Ub ligase in the embryonic cerebral cortex. The APC/C E3 Ub ligase ubiquitinates its substrates in conjunction with a limited set of E2 Ub-conjugating enzymes: UBE2S, UBCH10 (UBE2C) and, to a lesser extent, UBCH5 (UBE2D1) [82, 83]. As shown in Fig. 1D, *Ube2c* was the most highly expressed of these three E2 enzymes. Interestingly, *Ube2c* and *Cdc20* displayed similar patterns of expression (Fig. 6B). The decline in *Cdc20* expression mirrored the marked repression of *Ube2c* expression*.*
B.3. Fanconi anaemia (FA) E3 ligases
  The classification of the components of the Fanconi anaemia (FA) complex was established according to [85] and the Fanconi anaemia mutation database https://www2.rockefeller.edu/fanconi/). The FA complex is commonly described as a machine recruited to DNA lesions and playing a role in DNA repair. FANCL is the only protein of the FA complex displaying a ligase activity. However, no transcripts of its gene (*Fancl*) were found, suggesting poor or no activity under physiological developmental conditions. Of note, Ube2T, the E2 working in concert with FA E3 ligases, was also expressed at very low levels with TPM values decreasing from 17 to 4 between E11 and E17, and no *Ube2T* transcripts were detected on PN1 (Fig. 1D).
C. U-box RING E3 ligases:
  U-box RING E3 enzymes form another prominent class of E3 Ub ligases. They are characterized by a peculiar protein domain named the U-box and are structurally related to the RING finger family [86, 87]. U-box E3 Ub ligases are scaffolds that recruit a Ub-charged E2 and its colocalized substrate. Interestingly, mammalian U-box E3 Ub proteins interact with molecular chaperones or cochaperones such as Hsp90, Hsp70, DnaJc7, EKN1, CRN, and VCP [88]. U-box E3 Ub ligases can be found as monomers (i.e., UBE4) or homodimers (CHIP and PRPF19) [89]. Some U-box E3 Ub proteins have been identified as E4 enzymes due to their involvement in the assembly of poly-Ub chains on substrates that are first ubiquitinated by a non-U-box E3 Ub enzyme.
  Nine genes were analysed: *Stub1 (Chip), Prpf19 (Prp19), Ube4a (Ufd2b), Ube4b (Ufd2a), Ppil2 (Cyc4), Ubox5 (Uip5), Wdsub1, Act1 (Traf3ip2)* and *Aff4* (Fig. 7). Except for a transcript of *Act1*, transcripts of all U-box E3 Ub-encoding genes were found. No clear developmental regulation pattern was observed for the U-box ligases except for *Prpf19* (Prp19), the second most highly expressed U-box E3 Ub gene. The *Prpf19* TPM values decreased from 250 to 180 from E11 to PN1, a ⁓ 30% reduction in transcript abundance during corticogenesis. The functions of the *Prpf19* gene product are unknown, but it is an essential protein since mouse *Prpf19*-null mutants show lethality [90]. With TPM values of 300–350, the *Stub1 (Chip)* gene was the predominant gene in this group and one of the most highly expressed E3 Ub ligase genes. This high expression highlights its physiological relevance during brain formation and development. The U-box E3 Ub ligase CHIP can tag misfolded or damaged proteins for subsequent proteasomal degradation. A previously performed proteomic analysis identified hundreds of potential CHIP substrates in HEK 293 cells [91]. The very high level of *Stub1 (Chip)* expression underscores the physiological importance of CHIP during the protein quality control process and clearance of abnormal proteins throughout embryonic development.
  The UFD2a protein (coded by *Ube4b/Ufd2a)* has been found to be highly abundant in some brain areas, such as the cerebrum and cerebellum, of 8-week-old C57Bl6 mice [92]. Furthermore, immunohistochemical data have indicated that in the cerebral cortex, the UFD2a protein is localized mainly in the cytoplasm of neurons. Kaneko et al. [92] proposed that UFD2a contributes to the ubiquitination of specific substrates related to neuronal function. The high abundance of UFD2a proteins previously observed in the adult mouse brain differs from the low mRNA abundance of *Ube4b (Ufd2a)* transcripts in the embryonic brain.

**Table 1:** It gives the list of the genes encoding RING E3 ligases that were found to be weakly expressed during the formation of the cerebral cortex

E3 families	Genes	TPM values (mean values)				Expression pattern
		E11	E13	E17	PN1	
Cbl	*Cbl*	17.2	18.7	15.2	10.8	↘
	*Cblb*	4.9	4.5	5.1	3.1	=
	*Cblc*	n.d	n.d	n.d	n.d	
	*Cbll1*	20.7	23.5	30.2	23.0	↗
IAP	*Birc2*	23.5	29.7	24.1	17.0	↗
	*Birc3*	n.d	n.d	n.d	n.d	
	*Xiap*	18.4	19.9	17.8	16.0	=
	*Birc7*	n.d	n.d	n.d	n.d	
Listerin	*Ltn1*	12.7	14.8	14.3	12.0	=
Pellino	*Peli1*	11.0	15.6	24.2	14.7	↗
	*Peli2*	n.d	3.1	3.2	3.2	=
	*Peli3*	n.d	3.2	13.8	14.5	↗
Pex	*Pex2*	22.9	22.7	16.6	16.0	↘
	*Pex10*	17.3	20.6	17.1	11.6	↘
	*Pex12*	12.4	16.0	19.1	15.3	↗
Siah	*Siah1a*	14.1	16.5	23.2	22.5	↗
	*Siah1b*	36.6	31.0	13.0	9.2	↘
	*Siah2*	5.7	4.8	9.4	11.2	↗
	*Siah3*	n.d	2.7	n.d	n.d	
Ubr	*Ubr1*	4.4	5.4	7.3	5.6	=
	*Ubr2*	8.0	10.0	12.2	9.9	=
	*Ubr3*	7.9	8.4	11.4	11.8	↗
	*Ubr4*	19.0	22.4	27.8	27.0	↗
	*Ubr7*	61.4	58.3	35.2	27.7	↘

They all displayed TPM values < 65

n.d.: not detected, below the detection threshold. Depending on the gene, the abundance of transcripts increased, decreased or was nearly constant (↗, ↘ and =, respectively) during corticogenesis

**Table 2:** Gives an overview of the multi-subunit structure of CRLs and their modularity

Type of CRL	Cullin scaffold	RING-finger protein	Adaptor protein	Substrate recognition protein
CRL1	CUL1	ROC1 (Rbx1)	Skp1	F-box
CRL2	CUL2	ROC1 (Rbx1)	Elongin B/Elongin C	VHL-box
CRL3	CUL3	ROC1 (Rbx1)	BTB	
CRL4A	CUL4A	ROC1 (Rbx1)	DDB1	DCAF
CRL4B	CUL4B	ROC1 (Rbx1)	DDB1	DCAF
CRL5	CUL5	ROC2 (Rbx2)	Elongin B/Elongin C	SOCS-box
CRL7	CUL7	ROC1 (Rbx1)	Skp1	Fbw8
CRL9	CUL9 (PARC)	?	?	?

**Fig. 8:**
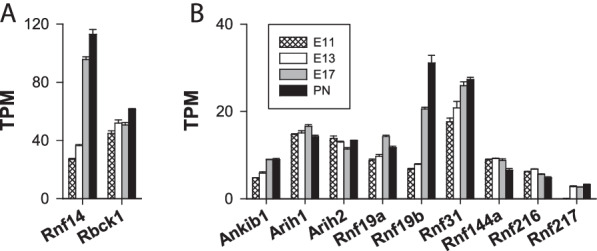
Expression of the genes encoding RBR E3 Ub ligases. The figure shows the predominant (**A**) and minor (**B**) RBR E3 Ub ligase genes

*RBR Ub ligases* The RBR Ub ligase family includes a few E3 Ub ligases. Fourteen RBR E3 genes were analysed (*Arih1, Arih2, Ankib1, Park2, Rbck1, Rnf14, Rnf19a, Rnf19b, Rnf31, Rnf144a, Rnf144b, Rnf216,* and *Rnf217*) (Fig. 8). The number of *Park2* and *Rnf144b* genes transcripts was lower than the detection limit. Notably, *Park2* encodes Parkin, a protein controlling mitophagy via the ubiquitination of mitochondrial proteins. Mitochondria can, however, be recycled via a Ub-independent pathway involving the specific autophagy receptors FUNDC1, BNIP3, and NIX [93]. Interestingly, the RNA-seq dataset indicated that, albeit sometimes at low levels, the *Fundc1*, *Bnip3* and *Nix* (*Bnip3l*) genes were all expressed with TPM values of ⁓ 10 (*Fundc1*), ⁓ 11 (*Bnip3*), and ⁓ 50 (*Nix*/*Bnip3l*). Based on these results, it is proposed that in the embryonic cerebral cortex, mitophagy is initiated independently of Parkin but via a Ub-independent process. As previously pointed out by [94], most of the data describing the regulation of mitophagy have been obtained with cells overexpressing Parkin and through the use of mitochondrial-depolarizing agents, which may not be relevant under the basal conditions of mitochondrial clearance.

*Rnf14* (ring finger protein 14, also known as Triad2), encoding transcriptional regulator RNF14, was the major RBR E3 gene expressed during cortex development. Its expression was positively regulated, with TPM values increasing from 27 to 113 from E11 to PN1 (Fig. 8A), representing a nearly > fourfold increase in *Rnf14* transcript abundance. RNF14 is an oncoprotein that promotes cell cycle progression and proliferation by inducing cyclin D1 expression [95]. In the developing (nontumorigenic) cerebral cortex, the expression of the cyclin D1 gene (*Ccnd1*) decreased from 333 to 32 TPM from E11 to PN1, a ⁓ tenfold reduction in *Ccnd1* transcript abundance. This decrease revealed an inverse relationship between the expression of the *Ccnd1* and *Rnf14* genes that contradicts the suggestion that RNF14 exerts a positive effect on cyclin D1 expression, at least under physiological conditions. The biological functions of RNF14 in the brain are unknown; however, our data indicated that RNF14 likely plays important functions in the cerebral cortex, particularly after the cessation of cell production (on E17 and onwards), during the maturation of neurons and the establishment of synaptic networks.

The other major RBR E3 gene in the embryonic cerebral cortex, although expressed at low levels, was *Rbck1*, encoding RanBP-type and C3HC4-type zinc finger containing 1 (HOIL-1 or HOIL-1L). Its TPM values were on the order of 45 at the onset of development and 60 at the latest stage, indicating a modest upregulation during corticogenesis (Fig. 8B). A recent report showed that RBCK1 plays a role in the linear ubiquitin assembly complex (LUBAC) [96] comprising the adaptor protein SHARPIN and two RBR E3 ligases: HOIP and HOIL-1L. HOIP is the main E3 catalytic centre of LUBAC and is necessary for linear ubiquitination. The gene encoding HOIP (*Rnf31*) was expressed at low levels (TPM values of 18–27) (Fig. 8B). HOIL-1L, which is the second most active and minor ligase of LUBAC, exerts a regulatory role in the complex by negatively regulating HOIL activity [96]. LUBAC is recruited to different protein aggregates associated with neurodegenerative diseases. LUBAC-dependent linear ubiquitination decreases the toxic potential of misfolded protein species and promotes their removal via the proteasome [97]. The linear ubiquitination catalysed by HOIP is antagonized by the DUB OTULIN [97]. Low HOIP and HOIL-1L levels in mice cause early embryonic lethality (on approximately E10.5) [98]. The other RBR E3 genes were also expressed at low levels, and their patterns of expression were not found to be developmentally regulated, except for *Rnf19b*, whose number of transcripts increased by a factor of ⁓5 between E11 and PN1, highlighting a putative function in neuronal development that remains to be discovered (Fig. 8B).

### Deubiquitinating enzymes (DUBs)

This section covers the gene expression of seven families of DUBs: USPs, UCHs, OTUs, MJDs, JAMMs, MINDYs and ZUP1 [3, 4]. DUBs are Ub hydrolases responsible of the deubiquitination process. Additionally, some of them such as USP5, UCH-L3, USP9X, USP7, and Otulin participate in the processing of Ub precursors [99]**.**

#### Ub-specific proteases (USPs)

**Fig. 9:**
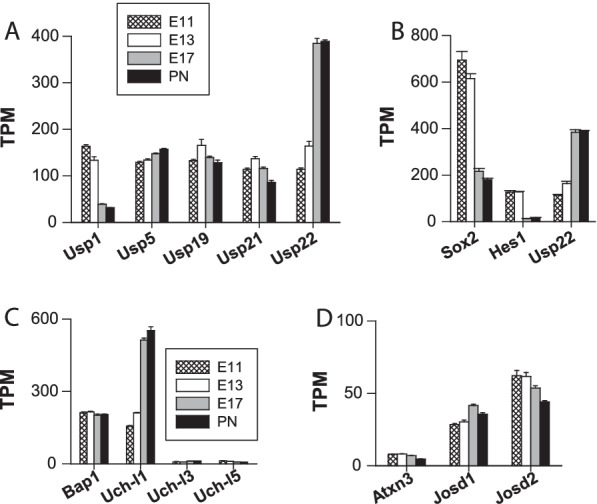
Expression of the *Usp*, *Uch* and *MJD* genes. This figure shows some of the major *Usp* genes (**A**), as well as *Uch* (**C**) and *MJD* (**D**) genes. Only the five most highly expressed *Usp* genes are reported in **A**. The other minor *Usp* genes can be found in Additional file 1: Fig. S3A and **B**. SOX2 and Hes1 proteins are two important targets of USP22, a predominant DUB in the cerebral cortex. **B** shows the normalized expression of the three respective genes (*Usp22*, *Sox2* and *Hes1*)

With approximately fifty members, USPs constitute the largest subfamily of DUBs [4]. In a recent report analysing the expression of *Usp* genes in the rat cerebellar cortex, only 32 USP-encoding genes were retained for analysis [100]. In the present study, we first selected fifty-four genes for analysis, but five of these genes (*Usp50, 39, 53, 54* and *Pan2*) showed no catalytic activity [18] and were therefore not analysed further. Ultimately, forty-nine genes were analysed. The transcripts of forty-five *Usp* genes were quantified, implying that four transcripts of *Usp* genes were undetected (*Usp13, Usp17, Usp18*, and *Usp26*). Large heterogeneity in gene expression was observed. For the sake of clarity, the five most highly expressed *Usp* genes are shown as a group in Fig. 9A, and the other members, with much lower transcript abundance, are shown in Additional file 1: Fig. S3A-B. Of note, USP9X was shown to play roles in neurodevelopment. However, a moderate level of gene expression was observed with TPM values ranging from 20 to 30 (Additional file 1: Fig. S3A). Furthermore, [101] found an age-related up-regulation of USP9X protein expression in the mouse brain with much higher protein levels in the adult brain, suggesting that USP9X could play important roles postnatally rather than during embryonic development.

With TPM values increasing from ⁓ 115 to ⁓ 390 between E11 and PN1, *Usp22* was the major gene and the most highly upregulated *Usp* gene (Fig. 9A). It was also one of the five most highly expressed DUB genes throughout corticogenesis. Its expression was developmentally regulated and showed a marked increase on E17. At this point, *Usp22* was the most highly expressed *Usp* gene and the second most highly expressed DUB gene after *Uch-l1* (see below). Our data were in line with previous reports showing high abundance of USP22 in the mouse embryonic brain [102, 103] and further indicating the specific expression of its gene in the cortex. USP22 proteins are critically required for embryogenesis since their loss leads to early embryonic lethality (at approximately E10.5) [103, 104]. USP22 proteins interfere with SOX2 and Hes1 activity, as well as that of other targets. *Sox2* is a pluripotency gene, and *Hes1* represses the expression of proneural genes, contributing to the regulated maintenance of neural/stem progenitor cells. In embryonic stem cells, an inverse correlation has been identified between SOX2 and USP22 protein levels [105]. Specifically, USP22 occupies the *Sox2* promoter and represses *Sox2* transcription [105]. Hes1, which undergoes a fast turnover rate due to its degradation by the proteasome, is deubiquitinated and stabilized by USP22 [102]. We therefore measured the expression of the *Sox2* and *Hes1* genes to gain further understanding of USP22-dependent regulatory mechanisms during corticogenesis. Interestingly, both genes were highly expressed when the *Usp22* expression was the lowest. In contrast, a profound increase in *Usp22* expression coincided with a marked reduction in *Sox2* and *Hes1* transcript abundance (Fig. 9B). Thus, a clear inverse correlation between *Usp22* (which promotes neuronal differentiation) and *Sox2* and *Hes1* (genes necessary for the maintenance of neural/stem progenitor cells) was identified.

*Usp1* was another highly expressed *Usp* gene for which a high abundance of transcripts was found on E11 and E13 (⁓ 160–130 TPM), corresponding to periods of intense cell division. *Usp1* was the second most highly expressed DUB gene on E11. The TPM values were ⁓ 40–30 on E17 and PN1 (Fig. 9A). This decrease in *Usp1* expression at E17 indicated that the gene may play a role in proliferation but not in the growth or maturation of neurons. In osteosarcoma cells, USP1 knockdown triggers osteogenic differentiation, whereas USP1 overexpression enhances proliferation, suggesting that, in this cell type, USP1 is involved in the maintenance of a stem cell state [106]. A similar finding was observed with glioblastoma cells [107]. The transcription of *Usp1* is regulated in a cell cycle-dependent manner, with transcription peaking during the S phase. The transcriptomic data clearly support the notion that *Usp1* is highly regulated during embryonic cortical development, showing high mRNA expression levels during stages of cell division and neurogenesis. Deletion of the *Usp1* gene has been associated with 80% perinatal lethality, and the surviving *Usp1*-deficient mice exhibited growth retardation [108].

In addition to *Usp1* and *Usp22*, *Usp5*, *Usp19* and *Usp21* were the other major *Usp* genes expressed in the embryonic cortical wall (Fig. 9A). However, these genes displayed no clear pattern of developmental expression. Similar to most DUBs, USP19 is a soluble cytosolic protein, but one prominent USP19 isoform possesses a C-terminal transmembrane domain, which enables its translocation to the endoplasmic reticulum (ER). USP19 seems to function in ER-associated degradation (ERAD). ER stress induction upregulates USP19 expression, and its biological relevance has been studied in muscle cells, where it plays a role in metabolic regulation and controls muscle mass [109]. Little is known regarding the neurobiological functions of *Usp19* and *Usp21*. The *Usp21* gene was one of the most highly *Usp* genes expressed during corticogenesis. Previously experiments conducted with embryonic stem cells showed that USP21 proteins control the balance between stem cell self-renewal and differentiation [110].

Notably, *Usp5* is continually highly expressed throughout embryonic development. Its protein product USP5, is primarily located in the cytosol and nucleoplasm, where it recognizes poly-Ub chains not conjugated to target proteins and contributes to maintaining the pool of free Ub monomers by removing Ub from the proximal end of these unanchored chains [111]. USP5, which is an important contributor of Ub precursors processing [99], has been studied extensively in relation to cancer, but this DUB is widely expressed. For example, USP5 has been shown to play a role in inflammatory and neuropathic pain by regulating the cell surface abundance of the Ca_v3.2_ protein, a T-type voltage-gated Ca^2+^ channel that plays important roles in nociception [112]. USP5 counterbalances the action of the E3 Ub ligase WWP1 [112]. Our data emphasize that certain components of the Ub system that are generally associated with cancers, such as USP5*,* are also highly expressed during development in nontumorous tissue.

As shown in Additional file 1: Fig S3A-B, many *Usp* genes were expressed in the cortical tissue throughout embryonic cortical development. For 10 *Usp* genes (*Usp4, 7, 9, 10, 14, 24, 28, 30, 36,* and *38*), the abundance of transcripts was nearly constant throughout corticogenesis, indicating that their expression was not developmentally regulated. In addition to *Usp1*, the expression of twelve genes was downregulated: *Usp3, 8, 21, 25, 37, 39, 40, 44, 45, 49, 51* and *54* (Additional file 1: Fig. S3A-B). Notably, *Usp25* and *Usp44* were only expressed at the beginning of corticogenesis. The expression of other *Usp* genes was upregulated, although to moderate levels. Interestingly, the expression of 4 *Usp* genes was induced at the end of corticogenesis (*Usp2, 29, 43,* and *53*), with no transcripts detected before E17. This finding points to a potential role of these DUBs in neuronal growth and the establishment of neural circuits, whereas the *Usp25* and *Usp44* gene products exert their biological effects during the neurogenesis period.

#### Ub carboxyl-terminal hydrolases (UCHs)

Four *Uch* genes, *Uch-l1, Uch-l3, Uch-l5* and *Bap1 (BRCA1-associated protein 1),* were expressed during corticogenesis, although the levels were considerably different (Fig. 9C). With TPM values ranging from ⁓155 (on E11) to ⁓550 (on PN1), *Uch-l1* clearly showed the highest expression levels in this subfamily, at least in the two latest stages. Its expression was markedly upregulated, with an abundance of transcripts increasing by a factor of 3.3 and 3.5 on E17 and PN1, respectively, compared to the abundance on E11. This observation is in line with the fact that UCH-L1 (also named PGP 9.5) is one of the most abundant brain proteins, representing up to 1–5% of total soluble brain proteins [113]. Isolated from brain extracts, UCH-L1 was originally described as a neuronal marker [113]. In a previous study on the brain, *Uch-l1* mRNA was detected in early stages of embryonic development [114] and was found in progenitor cells and neurons [115]. UCH-L1 has been postulated to facilitate neurogenesis and determine the morphology of progenitor cells [115]. The precise roles UCH-L1 plays in neuronal physiology are, however, poorly understood, but UCH-L1 dysfunction has been associated with several age-related neurodegenerative processes, such as Alzheimer’s and Parkinson’s diseases [116].

A high and constant abundance of *Bap1* transcripts was observed (TPM values of ⁓ 200) with no evidence of developmental regulation. *Bap1* was the most highly expressed *Uch* gene on E11-E13, whereas *Uch-l1* was the major gene expressed at the end of corticogenesis (E17-PN1) (Fig. 9C). The protein BAP1 was originally described as a nuclear DUB that exhibited tumour-suppressing properties. It regulates transcription and the DNA repair response. Additionally, BAP1 modulates intracellular Ca^2+^ signalling by deubiquitinating (and stabilizing) inositol 1,4,5-trisphosphate (IP3) receptors, prominent Ca^2+^ release channels in the ER [117]. Hence, BAP1 displays a basal prosurvival function by inhibiting the unfolded protein response induced by glucose deprivation [118]. Our data, together with results found in the literature, suggest that the protein product of *Bap1*, a highly expressed gene, plays important roles during the production, survival and differentiation of neural cells in the cortical wall during embryonic development.

#### MJD

The TPM values of the MJD genes *Atxn3*, *Josd1* and *Josd2* [4, 119] ranged from 4 to 60, revealing low to moderate transcript abundance (Fig. 9D). *Josd2* was the main MJD gene. Its expression was repressed throughout cortical development.

#### Otubain proteases (OTUs)

**Fig. 10:**
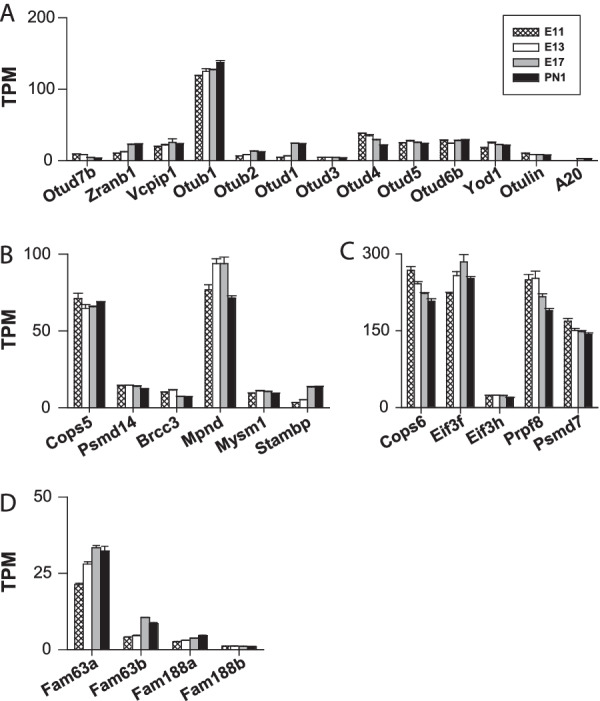
Expression of *Otus*, *JaMMs* and *MINDY* genes. Panels B and C show the expression of genes encoding JaMM proteins, which exhibit either enzymatic activity (**B**) or nonenzymatic activity (**C**). The other panels show the *Otu* gene expression levels (**A**) and *MINDY* gene expression levels (**D**)

The analysis encompassed fifteen genes, and for two of them, no transcript was found: *Otud6a* and *Otud7a* (*Cezanne2*). In addition to the *A20* gene (*Tnfaip3*) that was expressed exclusively (and at low level) on E17 and PN1, all the other *Otu* genes were expressed at low and moderate levels on the order of 5 to 30 TPM at all time points with no clear pattern of developmental regulation (Fig. 10A), except for *Otud1*. Its transcript abundance increased by nearly sixfold from E11 to PN1 (TPM values ranging from 4 to 23) (Fig. 10A). The gene *Otub1* was the only member of this family showing relatively high levels of expression (TPM values of 120–140). OTUB1 has been described as one of the most abundant DUBs in cells with ubiquitous tissue expression [18]. To date, the neuronal functions of *Otub1* have been poorly characterized. OTUB1 is found in the brain and is expressed in neurons but not in microglia or astrocytes [120]. OTUB1 attenuates the apoptosis of neuronal cells after intracerebral haemorrhage [120]. Hence, it is coenriched with α-synuclein [121]**,** the major component of Lewy bodies, which constitute a hallmark of Parkinson’s disease. The pathogenicity of OTUB1 has been underscored by [122], who showed that OTUB1 is an amyloidogenic protein that could contribute to the development of Parkinson’s disease. Notably, *Otud1*, although expressed at low levels (TPM values ranging from 4 to 23), was the most highly regulated gene of this subfamily; the abundance of *Otud1* transcripts increased nearly sixfold from E11 to PN1 (Fig. 10A).

#### Machado-Joseph disease protein domain proteases (JaMMs or Josephins)

First, we focused our analysis on the following seven JaMM genes: *Cops5 (Csn5), Psmd14, Brcc3, Mpnd, Mysm1, Stambp (Amsh),* and *Stambpl1*, which all encode DUBs with enzymatic activity. These genes were all expressed, except *Stambpl1* (Fig. 10B). *Cops5* and *Mpnd* were the major genes in this group, with TPM values of ⁓ 60 and ⁓ 70–90, respectively. For the other members, namely, *Psmd14, Brcc3, Mysm1*, and *Stambp (Amsh),* a low but continuous abundance of transcripts was observed (< 15 TPM) (Fig. 10B); however, among these genes, *Stambp* (*Amsh*) was found to be strongly and positively regulated, as indicated by a fourfold increase in transcript abundance between E11 and PN1 (from 3 to 14 TPM). Many JaMMs fail to display catalytic activity and are thus classified as pseudoenzymes [4]. The following pseudoenzyme genes were selected for analysis: *Cops6 (Csn6), Eif3f, Eif3h, Prpf8,* and *Psmd7*. As shown in Fig. 10C, they were all highly expressed. For instance, the TPM values of the 3 major genes *Cops6, Eif3f,* and *Prpf8* were on the order of 190–200. These transcripts were thus 3- to tenfold more abundant than the transcripts of the JaMMs genes *Cops5, Psmd14, Brcc3, Mpnd, Mysm1,* or *Stambp*. This finding indicates that the protein products of the *Cops6, Eif3f,* and *Prpf8* genes may exert important nonenzymatic biological functions in cells of the cortical wall during embryonic development.

#### Motif-interacting with Ub-containing novel DUB family (MINDY)

MINDY is a family of DUBs with four members: FAM63A (MINDY-1), FAM63B (MINDY-2), FAM188A (MINDY-3) and FAM188B (MINDY-4) [123]. The expression of these genes was investigated (Fig. 10D), and the TMP values were found to be on the order of ⁓ 2–10 for *Fam63b* and *Fam188a* and ⁓ 20–30 for *Fam63a*. No transcript of the *Fam188b* gene was found. Compared to that of the other DUB families, the lowest abundance of transcripts was found in this group of genes. The neuronal functions of members of the MINDY family are currently unknown.

#### ZUP1

ZUP1 (or ZUFSP, zinc finger with UFM1-specific peptidase domain) was identified as a seventh family of human DUB [124]. The murine *Zufsp* gene was expressed at extremely low levels (TPM values of 2–4, not shown). Nothing is known about the biological roles played by *Zufsp* in the rodent brain. In humans, the protein ZUFSP, which is mainly localized in the nucleus, is thought to be a putative DNA repair and/or replication factor involved in Ub signalling at DNA lesions [124].

## Conclusions

The contribution of the Ub system has been studied using various lines of embryonic stem cells and their differentiation into neural precursor cells. In contrast, in this study, no cell lines were employed, and data were extracted from an RNA-seq database [13], allowing us to detect transcriptomic changes in the core components of the Ub system during the formation of the cerebral cortex in mice. This strategy permitted us to describe the transcriptomic landscape of the whole tissue. This approach also revealed the large repertoire of functional components of the Ub system in embryogenesis. One important result indicated that the expression of Ub genes, notably *Ubb* and *Rps27a,* was extremely high. These two genes were among the 100 most highly expressed genes of the cortical wall. Our findings illustrate that the intricate ubiquitination network was governed by the E1 gene *Uba1*, which was more highly expressed, from 20- to 90-fold, than the other E1 gene *Uba6*. The most prominent E2 gene was *Ube2m*, encoding a Nedd8-conjugating E2 enzyme. The major Ub-conjugating E2 gene was *Ube2c*, the expression of which was profoundly downregulated during embryonic development. A large diversity of E3 Ub ligase gene transcripts was detected with distinct temporal patterns of expression. *Pja1, Trim67* (RING E3-encoding genes), *Stub1* (U-box E3-encoding gene), and *Nedd4* (HECT E3-encoding gene) were the most prominent E3 genes. A previous report analysed the expression of thirty DUB-encoding genes in the rat cerebellum, out of approximately one hundred DUB genes [100]**,** and thirty DUBs have also been independently described as being involved in the nervous system [119]. In this study, an extensive genome-wide gene expression analysis of the core components of the Ub machinery showed that more than 80 DUB genes were expressed during the formation of the cerebral cortex. This outcome provides a comprehensive survey of the large diversity of DUB gene expression and further indicates some important candidate products that may play major roles in cortex development. For instance, *Uch-l1* was one of the most highly expressed genes. It was also positively regulated during corticogenesis.

This study was based on a bulk transcriptomic analysis that did not discriminate between cell type or the cell lineages within the whole tissue sample. Moreover, certain cells, such as neurons, are highly polarized with several subcellular compartments (i.e., dendrites, cell body, axon) with distinct biological functions. Cellular polarity requires precise spatial targeting of the factors participating in the Ub pathway. In most (if not all) instances, the mechanisms governing the spatial targeting of Ub components are unknown. Despite these limitations, this study provides novel insights into the complex transcriptomic changes occurring during cerebral cortex formation.

One interest of the present work is the identification of several components of the Ub system known to be overexpressed in cancers that correspond to developmental genes highly expressed in the embryonic cerebral cortex under physiological conditions but are not related to tumour formation or progression, for instance *Ube2c* (E2 gene), *Trim28*, *Trim32*, and *Trim59* (E3 genes). The data collected may be used as a starting point for future functional studies of the rodent brain.

## Supplementary Information

**Additional file 1. Supplementary Figure 1**. It shows the expression of the minor genes (TPM values <40) encoding Ub-(A) and Ub-like (B) proteins conjugating E2 enzymes.** Supplementary Figure 2**. It shows the expression of the minor* Fbxw* (A),* Fbxl* (B) and* Fbxo* genes (TPM values <40).** Supplementary Figure 3**. It shows the expression of the minor *Usp* genes.

## Data Availability

The complete dataset is freely accessible on the GEO repository with the accession number GSE154677.
