# Supplementary figures and images for: Genome-wide analysis of genes encoding core components of the ubiquitin system during cerebral cortex development
Source: Mol Brain. 2022 Aug 16;15:72. doi: 10.1186/s13041-022-00958-z (PMC9380329; doi:10.1186/s13041-022-00958-z)

## Slide 1
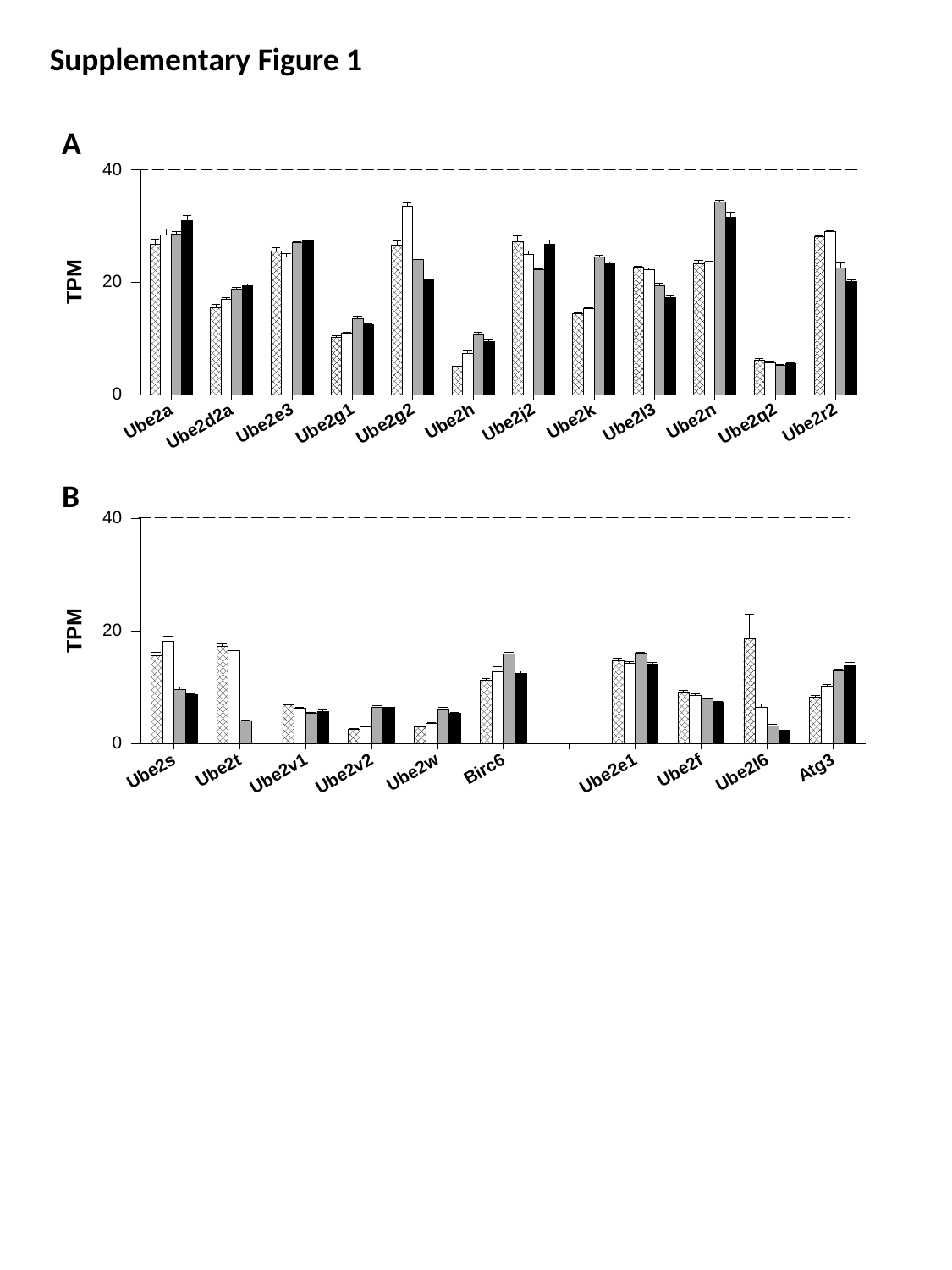

Supplementary Figure 1
A
B

## Slide 2
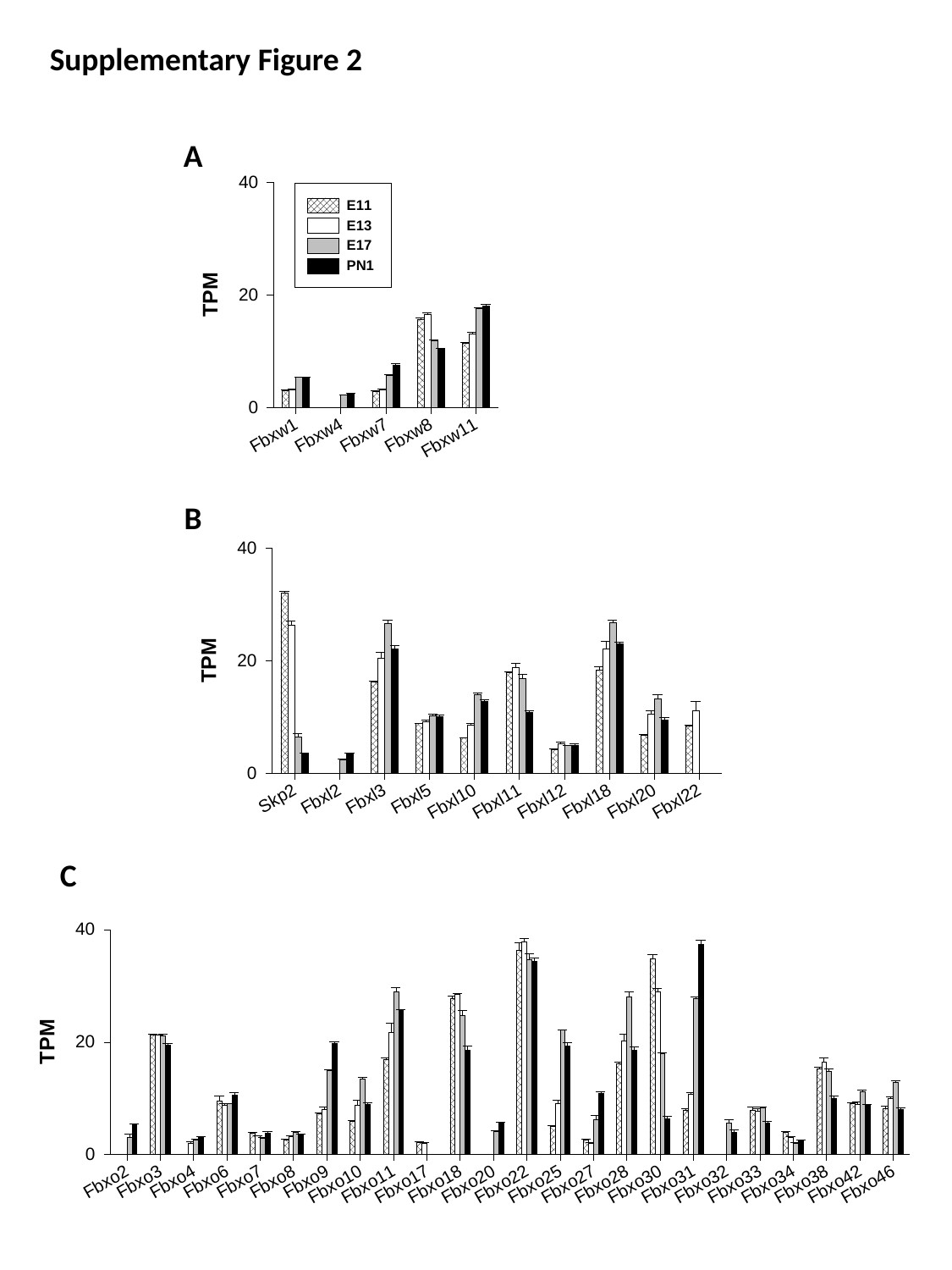

Supplementary Figure 2
A
B
C

## Slide 3
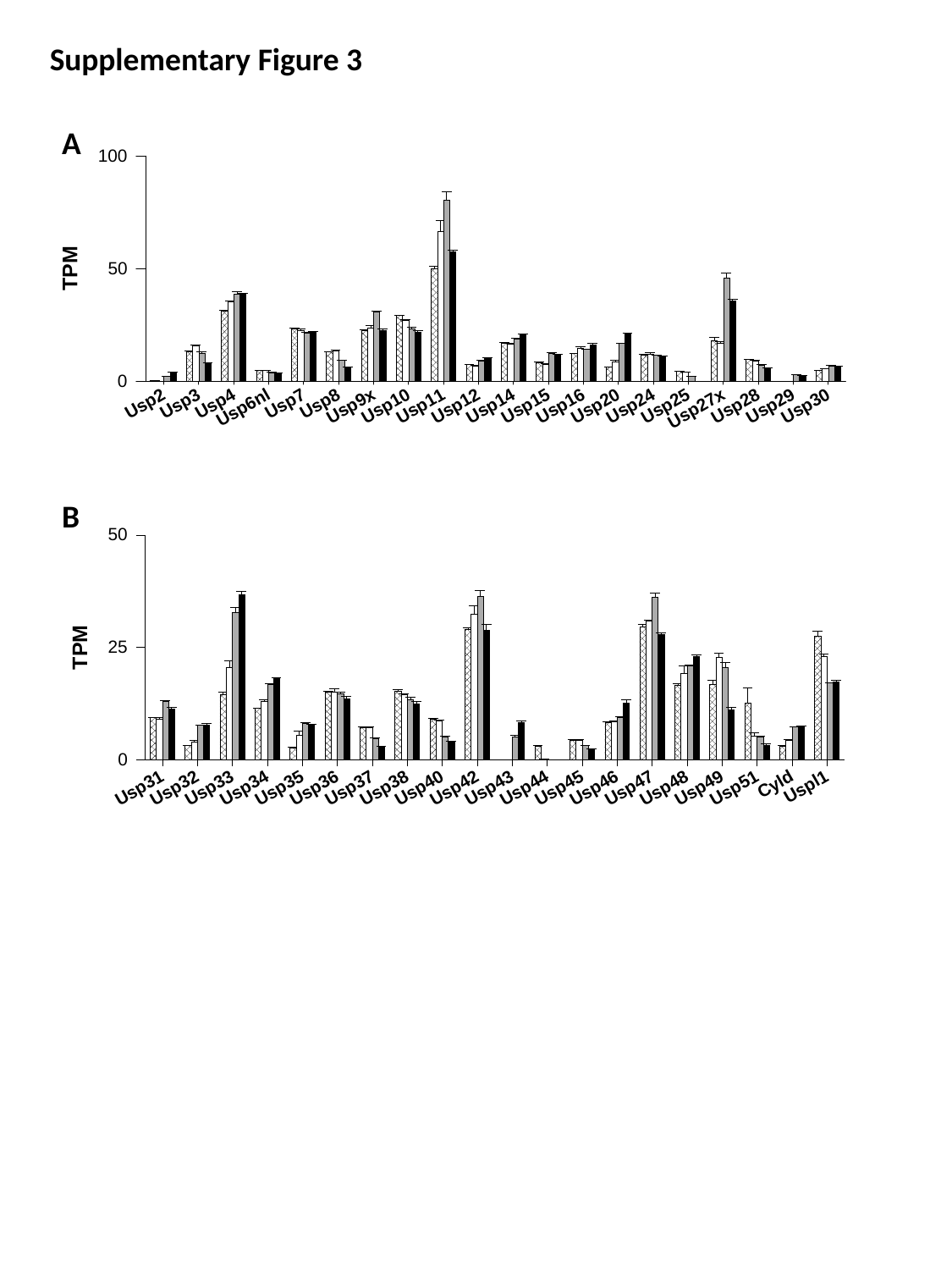

Supplementary Figure 3
A
B

Supplement: Supplementary file 1 — Additional file 1. Supplementary Figure 1. It shows the expression of the minor genes (TPM values <40) encoding Ub-(A) and Ub-like (B) proteins conjugating E2 enzymes. Supplementary Figure 2. It shows the expression of the minor Fbxw (A), Fbxl (B) and Fbxo genes (TPM values <40). Supplementary Figure 3. It shows the expression of the minor Usp genes. [file 13041_2022_958_MOESM1_ESM.pptx]
